# Effect of echinalkamide identified from *Echinacea purpurea* (L.) Moench on the inhibition of osteoclastogenesis and bone resorption

**DOI:** 10.1038/s41598-020-67890-x

**Published:** 2020-07-02

**Authors:** Bo Yoon Chang, Seul Ki Lee, Da Eun Kim, Jin Hye Bae, Thanh Tam Ho, So-Young Park, Mi Kyeong Lee, Sung Yeon Kim

**Affiliations:** 1 Institute of Pharmaceutical Research and Development, College of Pharmacy, Wonkwang University, Iksan, Jeonbuk 54538 South Korea; 2 College of Pharmacy, Chungbuk National University, Cheongju, 28160 Republic of Korea; 3 Department of Horticultural Science, Chungbuk National University, Cheongju, 28644 Republic of Korea

**Keywords:** Pharmacodynamics, Pharmacodynamics, Bone, Bone

## Abstract

Plant cell cultures have been exploited to provide stable production and new secondary metabolites for better pharmacological activity. Fractionation of adventitious root cultures of *Echinacea purpurea* resulted in the isolation of eleven constituents, including three new compounds. The structures of the three new compounds were determined to be an alkylamide (**1**), a polyacetylene (**2**) and a lignan (**3**) on the basis of combined spectroscopic analysis. To discover new types of antiresorptive agents, we screened for new compounds that regulate osteoclast differentiation, and survival. Among three new compounds, echinalkamide (compound **1**) had considerably inhibitory effects on RANKL-induced osteoclast differentiation, and on proliferation of osteoclasts and efficiently attenuated osteoclastic bone resorption without toxicity. In addition, echinalamide treatment inhibited the osteoclast—specific gene expression level. Echinalkamide achieved this inhibitory effect by disturbing phosphorylation of MAPK and activation of osteoclast transcription factors c-Fos and NFATc1. Conclusionally, our study investigated that echinalkamide remarkably inhibited osteoclast differentiation and osteoclast specific gene expression through repression of the MAPK–c-Fos–NFATC1 cascade.

## Introduction

*Echinacea purpurea* (L.) Moench is a perennial herb of the Astraceae family. This plant is mainly cultivated in North America and Europe and widely used as an herbal medicine and dietary supplement worldwide^1^. It is used in most popular herbal medicines for the treatment of common cold and respiratory disorders. Several Echinacea species have been shown to have various biological activities including immunomodulatory, anti-inflammatory, antiviral and antibacterial properties^2–4^. Due to its beneficial effects, investigations for the sufficient production of *E. purpurea* have been established in many fields^5,6^. Recently, in vitro tissue culture techniques with plant cells have been exploited to provide stable production. Moreover, culture conditions for improved biomass and bioactive metabolites accumulation are developed by regulating nutrients, elicitors and culture environments^7,8^. Plant cell cultures have recently been used to find new secondary metabolites for better pharmacological activity^9,10^. In vitro cultures including adventitious and hairy root cultures have been successively developed for the production of *E. purpurea* with improved biomass and metabolite accumulation^11–13^. Therefore, our present study was conducted to find new secondary metabolites from adventitious cultures of *E. purpurea*.

Previous studies have reported that *E. purpurea* reduces monocyte and macrophage responses to the major antigenic components of endotoxin, lipopolysaccharide, by inhibiting tumor necrosis factor (TNF)-α and prostaglandin E2 production. The immune-regulatory effect of E. purpurea is mediated through modulation of mitogen-activated protein kinases (MAPKs) and nuclear factor-kappa B (NF-κB) signaling pathways^14,15^.

We reasoned that candidate of osteoporosis treatment compound between components of could be identified on the basis of immune modulatory activity. In this study, we isolated new compounds that exhibited anti-inflammatory activity from the root of Echinacea. Among them, echinalkamide isolated from *E. purpurea* was chosen as a potent candidate compound for osteoporosis treatment. This study was designed to elucidate the effect of echinalkamide on inhibition of RANKL mediated osteoclasts differentiation and their mechanism.

## Material and method

### Reagents

Anti-NFATc1 (H-110), anti-c-Fos (H-125), anti-MMP-9, and anti-cathepsin K polyclonal antibodies were obtained from Santa Cruz Biotechnology Inc. (Europe, Germany). Anti-phospho-ERK1/2, -JNK, and -p38 polyclonal antibodies were obtained from Cell Signaling Technology (CA, USA). The RNA extraction kit (easy blue) was from Intron (Seongnam, Korea). The Taqman probes and one-step kit were obtained from Thermo Fisher Scientific (Massachusetts, USA). All other chemicals were obtained from Sigma Aldrich (St. Louis, USA).

### Plant material

Root of *E. purpurea* was cultured using Murashige & Skoog medium (MS 2.2 g/L, agar 8 g/L, pH 6.0) supplemented with indole butyric acid at 3% (w/v) of the total volume in Large-scale bioreactor^11^. A voucher specimen (CBNU201611-EPC) was deposited in the herbarium of College of Pharmacy, Chungbuk National University. The dried samples (1.3 kg) were extracted twice with 80% MeOH, which yielded the crude extract (432.7 g).

### Isolation of compounds

The crude extract was then suspended in H_2_O and partitioned successively with *n*-hexane (3.8 g), CH_2_Cl_2_ (24.5 g), EtOAc (4.7 g) and *n*-BuOH (26.5 g). The CH_2_Cl_2_ fraction was subjected to silica gel column chromatography with the mixture of CH_2_Cl_2_–MeOH to give 12 fractions (M1–M12). M6 was subjected to semipreparative HPLC with the mixture of MeOH–H_2_O to give 12 fractions (M6A–M6L). Compound **7** (1.5 mg) was obtained from M6A by Sephadex LH-20 column chromatography with *n*-hexane–CH_2_Cl_2_–MeOH (5:5:1), followed by semipreparative HPLC eluting with CH_3_CN–H_2_O. The M6B fraction was subjected to silica gel column chromatography with the mixture of MeOH–CH_2_Cl_2_ to give 3 fractions (M6B1–M6B3). Compound **6** (1.3 mg) was obtained from M6B2 by semipreparative HPLC eluting with MeOH–H_2_O. M6I was subjected to semipreparative HPLC with MeOH–H_2_O to give 5 fractions (M6I1–M6I5). Compound **3** (9.8 mg) was purified from M6I2 by semipreparative HPLC, using MeOH–H_2_O as eluent.

The EtOAc fraction was subjected to silica gel column chromatography with CH_2_Cl_2_–MeOH to give 15 fractions (E1–E15). Compound **2** (1.1 mg) was purified from E1 by semipreparative HPLC eluting with CH_3_CN–H_2_O. E2 was further subjected to Sephadex LH-20 column chromatography with MeOH (100%) to afford 8 subfractions (E2A–E2H). Compound **10** (2.3 mg) was purified from E2G by semipreparative HPLC eluting with CH_3_CN–H_2_O. Compound **8** (1.4 mg) was purified from E2F by semipreparative HPLC eluting with MeOH–H_2_O. E3 was chromatographed on Sephadex LH-20 eluting with MeOH (100%) to obtain 6 fractions (E3A–E3F). Compound** 9** (28.1 mg) was purified from fraction E3E by semipreparative HPLC eluting with CH_3_CN–H_2_O. Compound **5** (9.5 mg) was obtained from E3F by semipreparative HPLC eluting with CH_3_CN–H_2_O. Compound **4** (15.1 mg) was obtained from E5 by Sephadex LH-20 with CH_2_Cl_2_–MeOH (5:1), followed by semipreparative HPLC eluting with CH_3_CN–H_2_O. Fraction E6 was chromatographed on Sephadex LH-20 by using MeOH (100%) to give 4 fractions (E6A–E6D). Compound **1** (10.8) was purified from E6B by semipreparative HPLC eluting with CH_3_CN–H_2_O. Compound **11** (10.7 mg) was purified from E6C by semipreparative HPLC eluting with MeOH–H_2_O.

Echinalkamide (**1**). brown syrup; $\left[ \alpha \right]_{D}^{25}$-21.2 (c 0.03, MeOH); UV (MeOH) λ_max_ 261 nm; IR_max_ 3,364, 1646 cm^−1^; ^1^H NMR (500 MHz, CD_3_OD) δ 5.67 (1H, d, *J* = 11.5 Hz, H-2), 6.41 (1H, dd, *J* = 11.5, 11.5 Hz, H-3), 7.45 (1H, ddd, *J* = 15.5, 11.5, 1.0 Hz, H-4), 5.98 (1H, dt, *J* = 15.5, 6.5 Hz, H-5), 2.39 (2H, m, H-6), 2.43 (2H, m, H-7), 4.47 (2H, s, H-12), 3.03 (2H, d, *J* = 7.0 Hz, H-1′), 1.78 (1H, m, H-2′), 0.91 (6H, d, *J* = 7.0 Hz, H-3′, 4′), 4.41 (1H, d, *J* = 8.0 Hz, H-1″), 3.18 (1H, dd, *J* = 9.0, 8.0 Hz, H-2″), 3.26–3.38 (3H, m, H-3″, 4″, 5″), 3.84 (2H, m, H-6″) ppm; ^13^C NMR (125 MHz, CD_3_OD), δ 167.6 (C-1), 119.4 (C-2), 140.2 (C-3), 128.2 (C-4), 139.3 (C-5), 31.2 (C-6), 18.2 (C-7), 79.6 (C-8), 64.5 (C-9), 70.7 (C-10), 71.1 (C-11), 55.7 (C-12), 46.4 (C-1′), 28.3 (C-2′), 19.2 (C-3′, 4′), 100.9 (C-1″), 73.5 (C-2″), 76.6 (C-3″), 76.7 (C-4″), 70.2 (C-5″), 61.3 (C-6″) ppm; ESIMS (positive mode) *m/z* : 444 [M + Na]^+^; HR-ESI-MS (positive mode) *m/z*: 444.1992 (calcd for C_22_H_31_NNaO_7_^+^ 444.1993).

Echinacetylene (**2**). light brown syrup; $\left[ \alpha \right]_{D}^{25}$-14.5 (c 0.03, MeOH); UV (MeOH) λ_max_ 264 nm; IR_max_ 3,379, 1741 cm^−1^; ^1^H NMR (500 MHz, CD_3_OD) δ 4.31 (1H, t, *J* = 6.0 Hz, H-2), 2.78 (2H, m, H-3), 5.51 (1H, d, *J* = 10.8 Hz, H-8), 6.16 (1H, dq, *J* = 10.8, 7.2 Hz, H-9), 1.87 (3H, d, *J* = 7.2 Hz, H-10), 3.76 (OCH_3_) ppm; ^13^C NMR (125 MHz, CD_3_OD), δ 173.0 (C-1), 69.0 (C-2), 25.0 (C-3), 79.1 (C-4), 66.4 (C-5), 77.8 (C-6), 71.8 (C-7), 142.1 (C-8), 108.4 (C-9), 14.9 (C-10), 51.3 (OCH_3_) ppm; ESIMS: *m/z* 215 [M + Na]^+^; HR-ESI-MS: *m/z* 215.0679 (calcd for C_11_H_12_NaO_3_^+^ 215.0679).

Echisenecicariol (**3**). brown syrup; $\left[ \alpha \right]_{D}^{25}$-36.2 (c 0.03, MeOH); UV (MeOH) λ_max_ 272 nm; CD (1.7 × 10^−4^ M, MeOH) 248 nm (Δε + 3.53); IR_max_ 3,413, 1648 cm^−1^; ^1^H NMR (500 MHz, CDCl_3_) δ 6.67 (4H, s, H-2, 6, 2′, 6′), δ 4.97 (2H, d, *J* = 7.5 Hz, H-7, 7′), 2.52 (2H, m, H-8, 8′), 4.29 (2H, dd, *J* = 4.5, 2.5 Hz, H-9, 9′), 5.60 (2H, brs, H-11, 11′), 2.15 (6H, d, *J* = 1.0 Hz, H-13, 13′), 1.90 (6H, d, *J* = 1.0 Hz, H-14, 14′), 3,92 (12H, s, 4 × OCH_3_) ppm; ^13^C NMR (125 MHz, CD_3_OD) δ 132.6 (C-1, 1′), 102.7 (C-2, 2′), 147.1 (C-3, 3′, 5, 5′), 134.2 (C-4, 4′), 102.6 (C-6, 6′), 83.2 (C-7, 7′), 50.5 (C-8, 8′), 62.6 (C-9, 9′), 166.3 (C-10, 10′), 115.3 (C-11, 11′), 157.9 (C-12, 12′), 20.2 (C-13, 13′), 27.4 (C-14, 14′), 56.3 (4 × OCH_3_) ppm; ESIMS: *m/z* 623 [M + Na]^+^; HR-ESI-MS: *m/z* 623.2462 (calcd for C_32_H_40_NaO_11_^+^ 623.2463).

### Anti‑oxidant activities

The antioxidant efficacy of echinalkamide was evaluated by hydroxyl and superoxide radical scavenging assay. 2,2-diphenyl-1-picrylhydrazyl (DPPH, Sigma, CA, USA) was performed using the method described by Klouwen^16^. Vitamin C (50 µM, Sigma, CA, USA) was used as a positive control. Superoxide dismutase (SOD) assay was also performed using the SOD assay kit (Dojindo, Tokyo, Japan) according to the manufacturer’s instructions. Trolox (500 µg/Ml, Sigma, CA, USA) was used as a positive control.

### Bone marrow-derived macrophages (BMM) isolation

The protocol for mouse use in this experiment was approved by the Institutional Animal Care and Use Committee at Wonkwang University (Approval number WK18-112). All methods were performed in accordance with relevant guidelines and regulations. Bone marrow cells (BMCs) were isolated from the long bones of 6-week-old C57BL6 mice by flushing with α-MEM containing antibiotics and red blood cells (RBCs) were removed using RBC lysis buffer.

### Cell viability

RAW264.7 cells were obtained from ATCC (TIB-71™, Virginia, USA). A monolayer of cultured RAW264.7 cells was trypsinized and the cell count adjusted to 1.0 × 10^5^ cells/mL using DMEM containing 10% FBS. The cells were then cultured for 24 h in 24-well culture plates at 2.5 × 10^4^ cells/well in the presence or absence of 1, 5, 10, 50, 100 µM compound 1–3 or 10 µg/mL LPS. After incubation for 1 days, 100 µL of 3-(4,5-dimethylthiazol-2-yl)-2,5-diphenyltetrazolium bromide (1 mg/mL MTT, Sigma Aldrich, St. Louis, USA) reagent was added to each well and incubated for 4 h. The supernatant containing the MTT solution was discarded, and then the MTT formazan crystals formed in the cells were dissolved in dimethyl sulfoxide (DMSO, Sigma Aldrich, St. Louis, USA). Absorbance was measured at 520 nm by using an enzyme-linked immunosorbent assay (ELISA, Tecan, Switzerland) plate reader.

BMMs (1 × 10^4^ cells/well) were seeded in triplicate in 96-well plates in Minimum Essential Medium Eagle Alpha Modification (α-MEM, Gibco, Waltham, Massachusetts, USA) and then incubated for 3 days with macrophage colony stimulating factor (M-CSF, 30 ng/mL, Sigma Aldrich, St. Louis, USA) in the presence or absence of various concentrations of echinalkamide. After 4 days, cell viability was measured using the MTT assay.

### Anti-inflammation activity

Inhibitory effect of compounds on LPS-induced NO production and TNF-α secretion was assessed using RAW264.7cell^17^. RAW 264.7 cells were treated with 10 μg/ml lipopolysaccharide (LPS) in the presence or absence of compound **1**–**3**. After 24 h incubation, the amount of Nitric oxide (NO) and Tumor necrosis factor-alpha (TNF-α) production was measured according to previous report^18^.

### Osteoclast differentiation and TRAP staining

BMCs were cultured for 4 days in the presence of M-CSF (30 ng/mL) to differentiate into BMM. To investigate the effect of differentiation of echinalkamide on osteoclast differentiation, BMMs with the various concentration of echinalkamide in the presence of M-CSF (30 ng/mL) and RANKL (100 ng/mL) in 96-well plates were processed. After 4 days, the cells were fixed with formalin, stained with TRAP, and then multinuclei (more than 3) were counted as osteoclasts.

### Real-time PCR

Total RNA was extracted from harvested cells using Easy blue (Intron, Seongnam, Korea). The amount of RNA was quantified with nanodrop (Thermofier, Massachusetts, USA). Each reaction mix (Applied Biosystems, California, USA) contained between 50 and 100 ng of RNA in a total reaction volume of 25 μL. Probes for the quantitative amplification of Complete information for FOS (c-FOS, Mm00487425, Applied Biosystems, California, USA), Nuclear factor of activated T-cells, cytoplasmic 1 (NFATc1, Mm00479445, Applied Biosystems, California, USA), cathepsin K (Mm01255862, Applied Biosystems, California, USA), Matrix metallopeptidase 9 (MMP9, Mm00600164, Applied Biosystems, California, USA), and glyceraldehyde 3-phosphate dehydrogenase (GAPDH, Mm03302249, Applied Biosystems, California, USA) were validated using TaqMan Gene Expression Assay (Applied Biosystems, California, USA). Conditions for real-time quantitative RT-PCR were as follows: 30 min at 48 °C, 10 min at 95 °C (RT inactivation and initial activation), and then 40 cycles of amplification for 15 s at 95 °C (denaturation) and 1 min at 60 °C (annealing and extension). Data analysis was performed using SDS 2.1.1 software. To normalize expression to that of the GAPDH housekeeping gene, a mathematical model of partner expression ratio including PCR efficiency was applied to sample quantification.

### Western blot analysis

Treated osteoclasts were harvest and lysed by direct addition of a lysis buffer (containing protease inhibitor and phosphatase inhibitor cocktails, Intron, Seongnam, Korea). The nuclear/cytosol fractionation kit (Bio Vision Technology Inc., New Minas, NS, Canada) was used to separate nuclear and cytoplasmic proteins according to the manufacturer’s protocol. After the proteins were isolated, the concentration of the samples was determined using a bicinchoninic acid (BCA) assay kit (Thermo Fisher Scientific Inc., Massachusetts, USA). Sample (20 μg) per lane were electrophoresed on a 12% reducing SDS–PAGE gel and transferred onto a nitrocellulose membrane (Biorad, California, USA). The membrane was blocked with 5% skim milk and sequentially incubated with anti-c-FOS, anti-NFATc1, anti-MMP-9, anti-cathepsin K, and anti-actin antibodies at 4 °C overnight (all antibodies were used at a 1:1,000 dilution and were purchased from Cell Signaling Technology. Specific protein bands were visualized using horseradish peroxidase-conjugated secondary antibodies (1:1,000 dilution, Enzo Life Sciences, Lausen, Switzerland) followed by Enhanced chemiluminescence (ECL) detection (Amersham Pharmacia Biotech, Piscataway, NJ, USA). Protein images were captured using the FluorChem E image system (ProteinSimple, Santa Clara, CA, USA). Quantify images of western blot bands was measured using Image J software (NIH, Maryland, USA).

### Osteoclastic resorption

Osteoclastic resorption assay plates (Corning, New York, USA) were incubated with the indicated doses of echinalkamide in the presence of M-CSF (30 ng/mL, Sigma-aldrich St. Louis, USA) and RANKL (100 ng/mL, Sigma-aldrich St. Louis, USA) on 7 days. To quantify resorption, the cells were removed with 10% NaClO (Sigma-aldrich St. Louis, USA) and the wells were washed with water and air dried. Resorption area was measured using Image J software (NIH, Maryland, USA).

### Statistical analysis

Data are expressed as mean ± SD values. All the data were confirmed by technical replicated (n = 3). Significant differences were compared using repeated measures ANOVA followed by the Newman-Keuls multiple range test. Statistical significance was defined as *P* < 0.05. All statistical analyses were performed using GraphPad Software. Inc. (San Diego, CA).

## Results

### Chemistry

**Figure 1:**
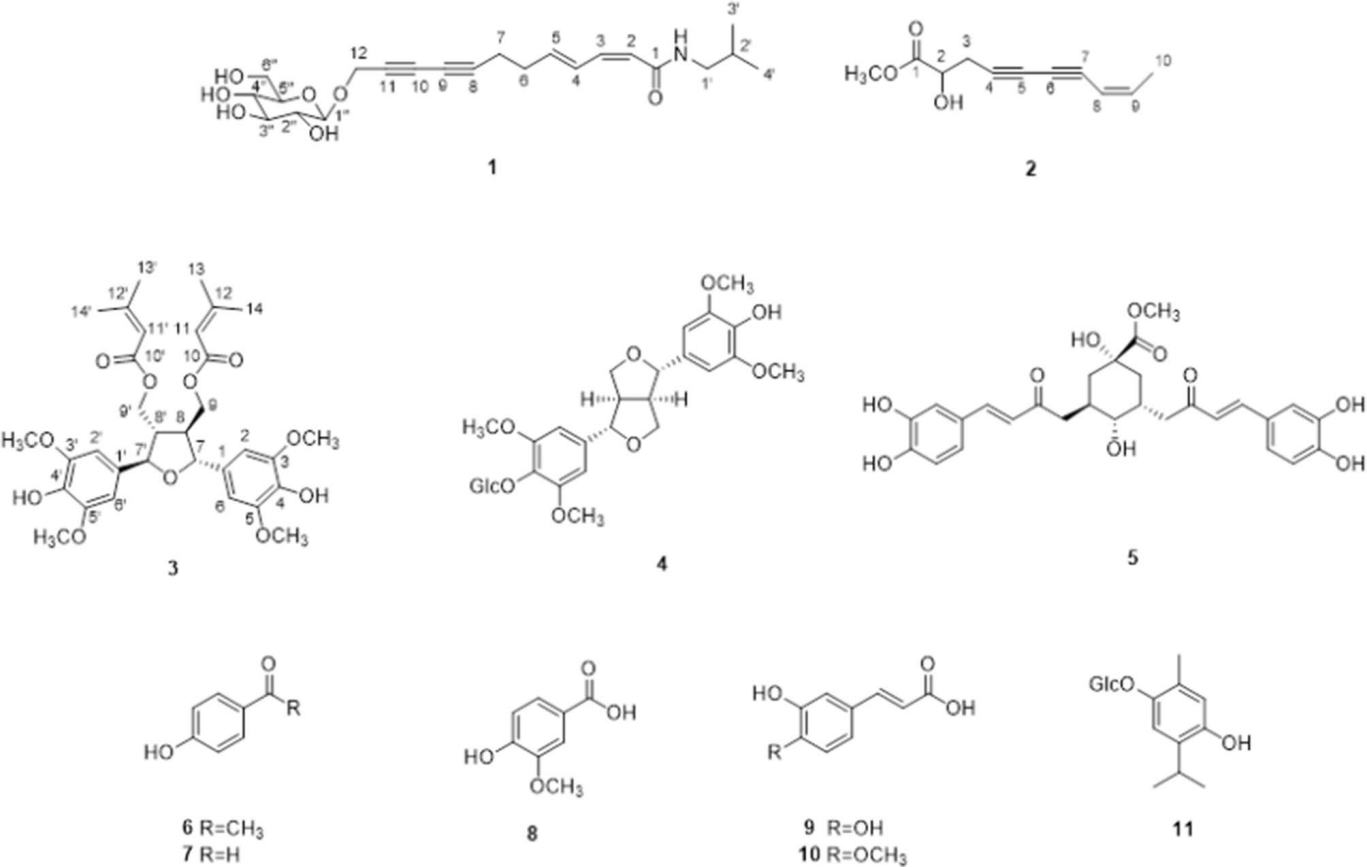
Chemical structures of compounds **1**–**11** from adventitious root cultures of *E. purpurea.*

Fractionation and separation of the adventitious root cultures of *E. purpurea* yielded eleven compounds including three new compounds (Fig. 1). The eight known compounds were identified as (+)-syringaresinol *O*-*β*-d-glucopyranoside (**4**), 3,5-di-*O*-caffeoylquinic acid methyl ester (**5**), 1-(4-hydroxyphenyl)-1-ethanone (**6**), benzaldehyde (**7**), benzoic acid (**8**), cinnamic acid (**9**), 4-methoxycaffeic acid (**10**), and thymoquinol 2-*O-β*-glucopyranoside (**11**), by the analysis of their spectroscopic data and comparison with literature values^19–21^.

Compound **1** was isolated as brown syrup. Its molecular formula was determined as C_22_H_31_NO_7_ based on the HR-ESI-MS ion at *m/z* 444.1992 ([M + Na]^+^, calcd 444.1993) and ^13^C NMR data. The IR spectrum showed typical absorption bands of hydroxy and carbonyl groups at 3,364 and 1646 cm^−1^, respectively. The ^1^H and ^13^C NMR spectrum suggested the presence of a sugar moiety from the characteristic anomeric proton at δ_H_ 4.41 (1H, d, *J* = 8.0 Hz, H-1″) and six glucosyl oxycarbons at [δ_C_ 100.9, 76.7, 76.6, 73.5, 70.2, 61.3]. The sugar was identified as a glucopyranose based on the coupling constants and chemical shifts of the protons and carbons^22,23^. The configuration of a glucose moiety was determined to be β form based on the coupling constant of *J* = 8.0 Hz^24^. The signals at [δ_H_ 3.03 (2H, d, *J* = 7.0 Hz, H-1′), 1.78 (1H, m, H-2′) and 0.91 (6H, d, *J* = 7.0 Hz, H-3′, 4′); δ_C_ 46.4 (C-1′), 28.3 (C-2′), 19.2 (C-3′, 4′)] in the ^1^H and ^13^C NMR spectrum together with the HMBC correlations between H-3′/C-4′, H-3′/C-1′ and H-3′/C-2′ suggested the presence of an isobutyl group^19^. Also, the ^1^H NMR data showed the signals of two pairs of olefinic protons at δ_H_ 5.67 (1H, d, *J* = 11.5 Hz, H-2) and 6.41 (1H, dd, *J* = 11.5, 11.5 Hz, H-3) as well as δ_H_ 7.45 (1H, ddd, *J* = 15.5, 11.5, 1.0 Hz, H-4) and 5.98 (1H, dt, *J* = 15.5, 6.5 Hz, H-5)] in *Z* and *E* configurations, respectively, as determined by the coupling constants of *J* = 11.5 Hz and 15.5 Hz. In addition, two methylene signals at δ_H_ 2.39 (2H, m, H-6) and 2.43 (2H, m, H-6) and one oxymethine signal at δ_H_ 4.47 (2H, s, H-12) were observed in the ^1^H NMR spectrum. The ^13^C NMR spectrum showed signals for two pairs of olefinic carbons, two methylenes and one oxymethylene, consistent with ^1^H NMR data. In addition, four quaternary carbons at δ_C_ 79.6, 64.5, 70.7 and 71.1 in ^13^C NMR and HSQC spectrum were assigned as two pairs of triple bonds. The ^1^H and ^13^C NMR data for **1** were very similar to those of dodeca-2*Z*, 4*E*-diene-8,10-diynoic acid isobutylamide^24^, but oxymethylene and glucose moieties were appeared instead of a methyl group. Therefore, **1** was determined as a 12-*O*-β-glucopyranoside of 12-hydroxydodeca-2*Z*-4*E*-diene-8,10-diynoic acid isobutylamide, and named echinalkamide.

Compound **2** was isolated as a light brown syrup. The molecular formula of **2** was determined as C_11_H_12_O_3_ from the HR-ESI-MS *m/z* 215.0679 ([M + Na]^+^, calcd 215.0679) and ^13^C NMR data. The IR spectrum showed absorption bands for hydroxy (3,379 cm^−1^) and carbonyl (1741 cm^−1^) groups. In the ^1^H NMR spectrum, the signals at δ_H_ 5.51 (1H, d, *J* = 10.8 Hz, H-8), and 6.16 (1H, dq, *J* = 10.8, 7.2 Hz, H-9) suggested the presence of olefin in *cis*-form. The ^1^H NMR data also showed the presence of an oxymethine at δ_H_ 4.31 (1H, t, *J* = 6.0 Hz, H-2), a methylene at δ_H_ 2.78 (2H, m, H-3), a methyl at δ_H_ 1.87 (3H, d, *J* = 7.2 Hz, H-10) and a methoxy group at δ_H_ 3.76. The ^13^C NMR showed signals corresponding to the aforementioned ^1^H NMR data such as olefinic carbons at δ_C_ 142.1 and 108.4, an oxymethine at δ_C_ 69.0, a methylene at δ_C_ 25.0, a methyl at δ_C_ 14.9 and a methoxy at δ_C_ 51.3, as confirmed by HSQC analysis. Also, the presence of a carbonyl and two sets of triple bonds were deduced by the signals at δ_C_ 173.0 as well as signals at δ_C_ 79.1, 66.4, 77.8, 71.8, respectively. The HMBC correlation correlations between H-3 and C-1, C-2, C-4, C-5, between H-9 and C-7, C-10 together with the correlation between OCH_3_ and C-1 revealed the methyl ester at C-1, which indicated the hydroxyl group at C-2. Taken together the structure of **2** as shown and named echinalcetylene.

**Figure 2:**
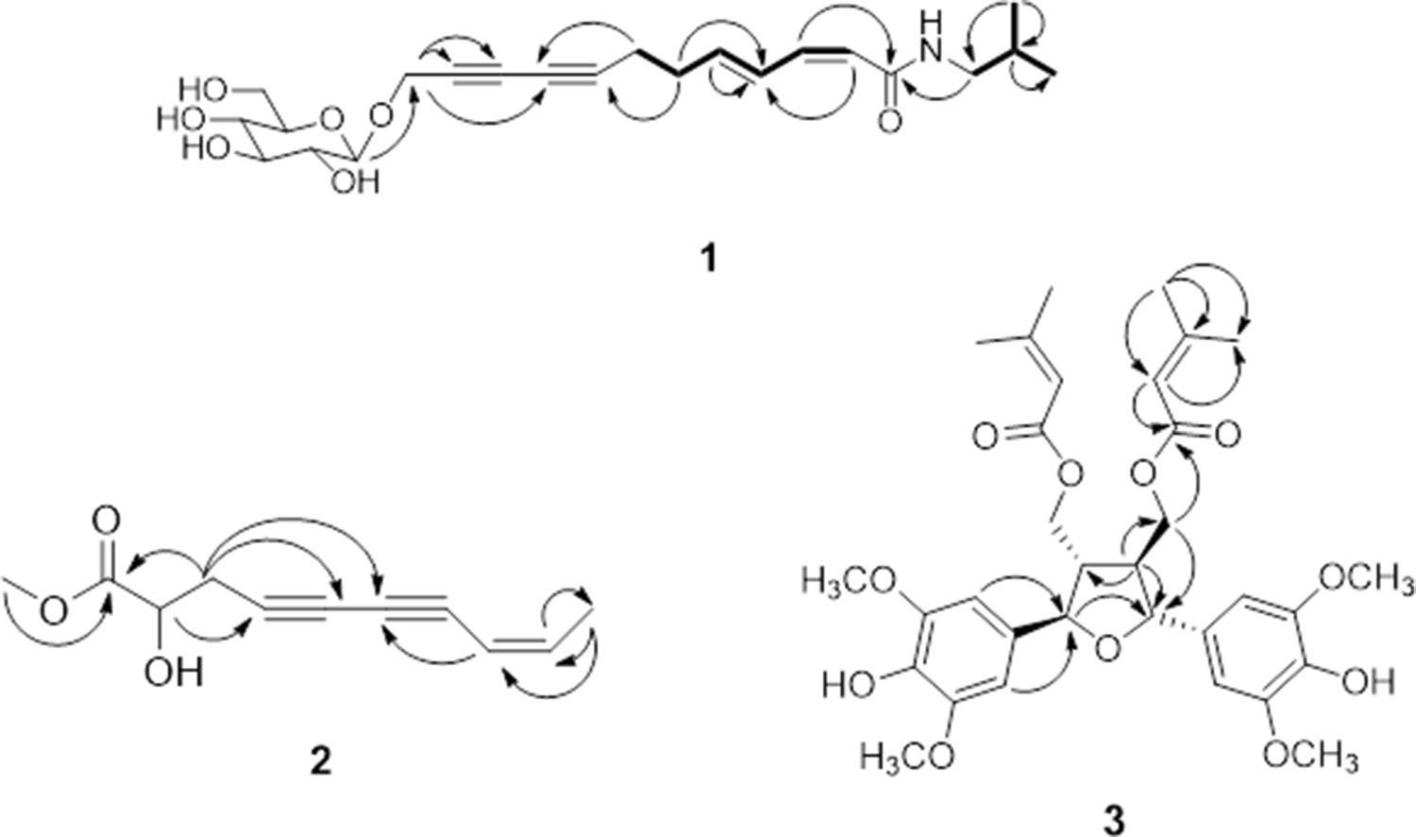
Key HMBC (→) and COSY (▬) correlations of new compounds **1**–**3**.

Compound **3** was obtained as a brown syrup. The molecular formula of C_32_H_40_O_11_ was determined by the HR-ESI-MS ion at *m/z* 623.2462 ([M + Na]^+^, calcd 623.2463). Contrary to our expectation of molecular formula from HR-ESI-MS, the ^13^C NMR spectrum showed only 13 carbon resonances, suggesting **3** is a symmetrical molecule. Detailed analysis of the ^1^H and ^13^C NMR spectra deduced the presence of senecioyl moieties at δ_H_ 5.60 (2H, brs, H-11/11′), 2.15 (6H, d, *J* = 1.0 Hz, CH_3_-13/13′) and 1.90 (6H, d, *J* = 1.0 Hz, CH_3_-14/14′); δ_C_ 166.3 (C-10/10′), 115.3 (C-11/11′), 157.9 (C-12/12′), 20.2 (C-13/13′), and 27.4 (C-14/14′), which were confirmed by HMBC correlations from H-11/C-10, H-11/C-13 and H-11/C-14^25^. Besides the signals attributed to the two senecioyl moieties, four aromatic protons at δ_H_ 6.67 (4H, s, H-2/2′, 6/6′), two oxymethines at δ_H_ 4.97 (2H, d, *J* = 7.5 Hz, H-7/7′), two methines at δ_H_ 2.52 (2H, m, H-8/8′), two oxymethylenes at δ_H_ 4.29 (2H, dd, *J* = 4.5, 2.5 Hz, H-9/9′) and four methoxy at δ_H_ 3.92 (12H, s) were observed in the ^1^H NMR spectrum. The ^13^C NMR spectrum showed the signals at δ_C_ 50.5 (C-8/8′), 62.6 (C-9/9′), 83.2 (C-7/7′) and 56.3 (OCH_3_) as well as two aromatic carbons at δ_C_ 132.6 (C-1/1′), 102.7 (C-2/2′, 6/6′), 147.1 (C-3/3′, 5/5′) and 134.2 (C-4/4′), consistent with ^1^H NMR data. The correlations at H-2/C-7, H-6/C-7, H-8/C-7 and H-8/C-9 were observed in the HBMC spectrum. The HMBC spectrum also showed the correlation between H-7 and C-7′. Based on these data, compound **3** was suggested as icariol A2 with two senecioyl moieties in symmetric structure. The senecioyl groups were placed at C-9/9′ by the correlations between H-9/9′ and C-10/10′ in the HMBC spectrum (Fig. 2). The relative stereochemistry was determined by NOESY analysis from the correlation between H-7 and H-9. The absolute configuration at the C-7 position was assessed by the circular dichroism (CD) analysis. The CD spectrum of **3** showed a positive Cotton effect at 248.0 nm (Δε + 3.53), thus the configuration of C-7 of **3** was elucidated as *R* by the comparison with the negative Cotton effect at 246.3 nm (Δε − 4.4) of 7*S* configuration of 7*S*,7′*S*,8*R*,8′*R*-icariol A2-9-*O-β*-_D_-glucopyranoside^26^. Based on these data, the structure of compound **3** was defined as shown, and the compound was named echisenecicariol.

### Effect of newly isolated compounds on antioxidant activity

**Figure 3:**
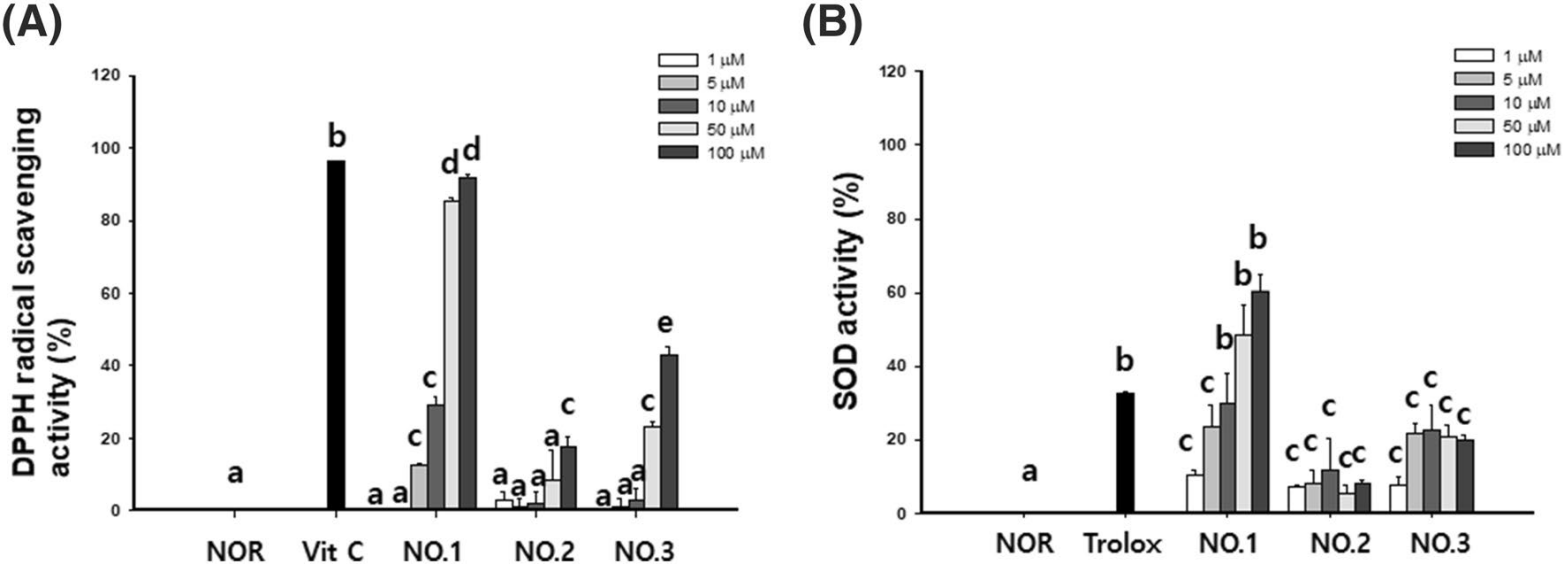
Antioxidant activity of new compounds **1**–**3**. (**A**) Hydroxyl radical scavenging and (**B**) SOD activity of new compounds **1**–**3**. All the data were confirmed by technical replicated (n = 3). The results are presented as the mean ± SD. Values with different letters (a, b, c, d, e) are significantly different one from another (one-way ANOVA followed by Newman–Keuls multiple range test, *p* < 0.05).

The antioxidant activity of compounds **1**–**3** was evaluated by measuring the DPPH and SOD activities. As shown in Fig. 3, compound **1** showed scavenging activity against DPPH and SOD similar than positive controls (DPPH: vitamin C, SOD: trolox).

Among the three compounds, compound 1 showed the highest antioxidant effect. In detail, compound 1 showed a concentration-dependent increase in DPPH radical scavenging activity from 5 μM (12.4 ± 2.2%), and a similar increase from 50 μM (83.4 ± 5.1%) to vitamin c 50 μM (90.2 ± 2.8%) SOD activity increased in a concentration-dependent manner, and compound 1 showed an increase similar to that of trolox at 500 μg/mL(35.1 ± 2.0) from 50 μM treatment(49.2 ± 9.4).

### Effect of newly isolated compounds on anti-inflammatory activity

**Figure 4:**
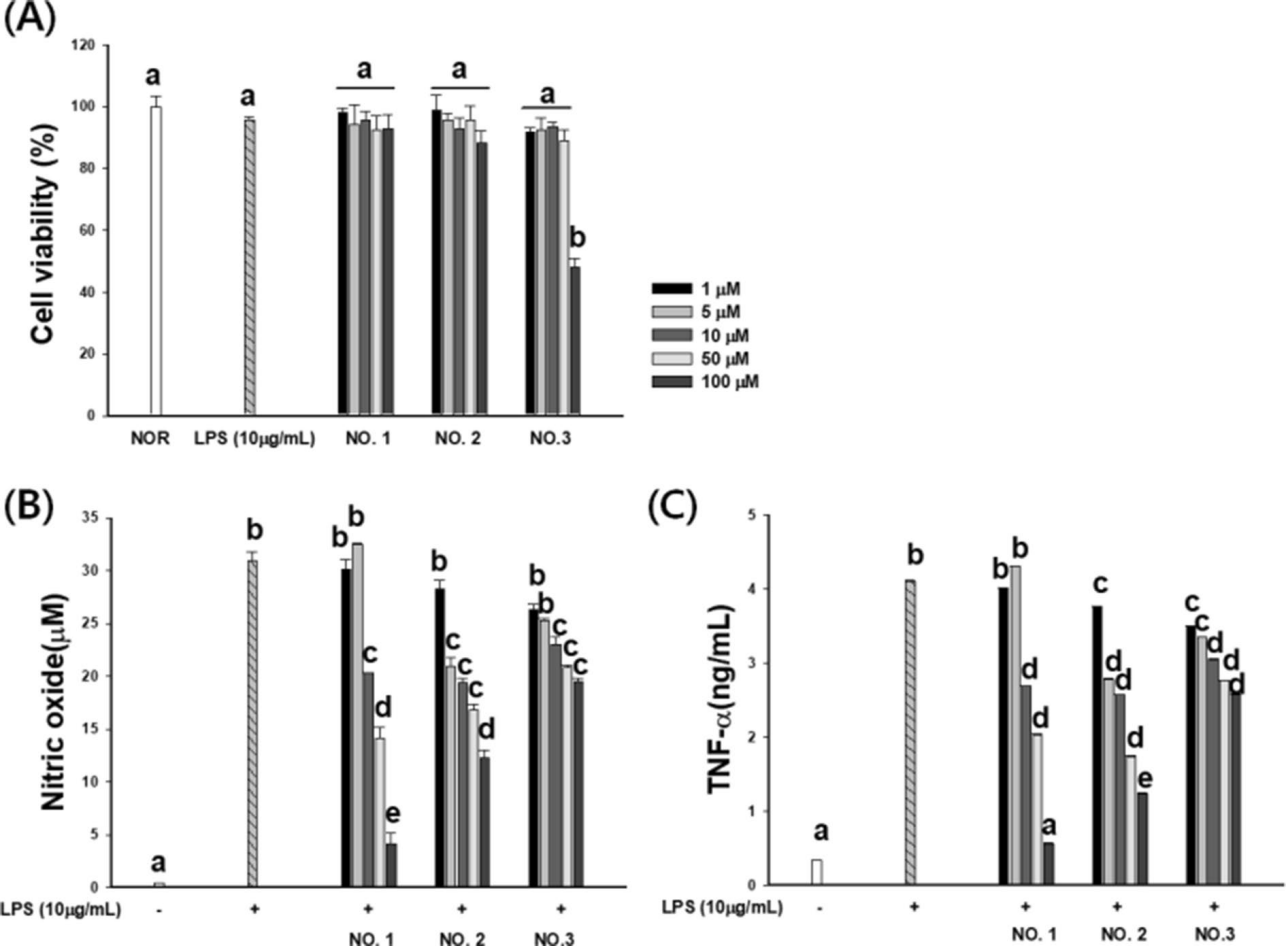
Effect of new compounds **1**–**3** on the production of NO and TNF-α in LPS-stimulated RAW 264.7 cells. (**A**) RAW264.7 cells were treated with new compounds **1**–**3** or 10 µg/mL LPS for 24 h, after which, cell viability was measured using an MTT assay. Cells were treated new compounds **1**–**3** for 1 h before incubation with LPS for 24 h. Culture supernatants were then isolated, and the amounts of (**B**) NO and (**C**) TNF-α production were determined. All the data were confirmed by three repeated tests. The results are presented as the mean ± SD. Values with different letters (a, b, c, d, e) are significantly different one from another (one-way ANOVA followed by Newman–Keuls multiple range test, *p* < 0.05).

We investigated the anti-inflammatory effects of newly isolated compounds by measuring the production of NO and TNF-α in LPS-stimulated RAW 264.7 macrophages. The cytotoxic effects of each compound were also tested to ensure that the inhibitory effect on NO production was whether it was due to cell death. Compounds **1** and **2** dose-dependently reduced NO and TNF-α production stimulated by LPS without any significant cytotoxic effects at the concentration ranging from 1 to 100 μM. However, compound **1** reduced NO production and displayed cytotoxic effects at 100 μM. Compounds **1**–**3** conclusively showed anti-inflammatory activity probably by interfering with NO and TNF-α production (Fig. 4).

### Effect of echinalkamide on cell viability

**Figure 5:**
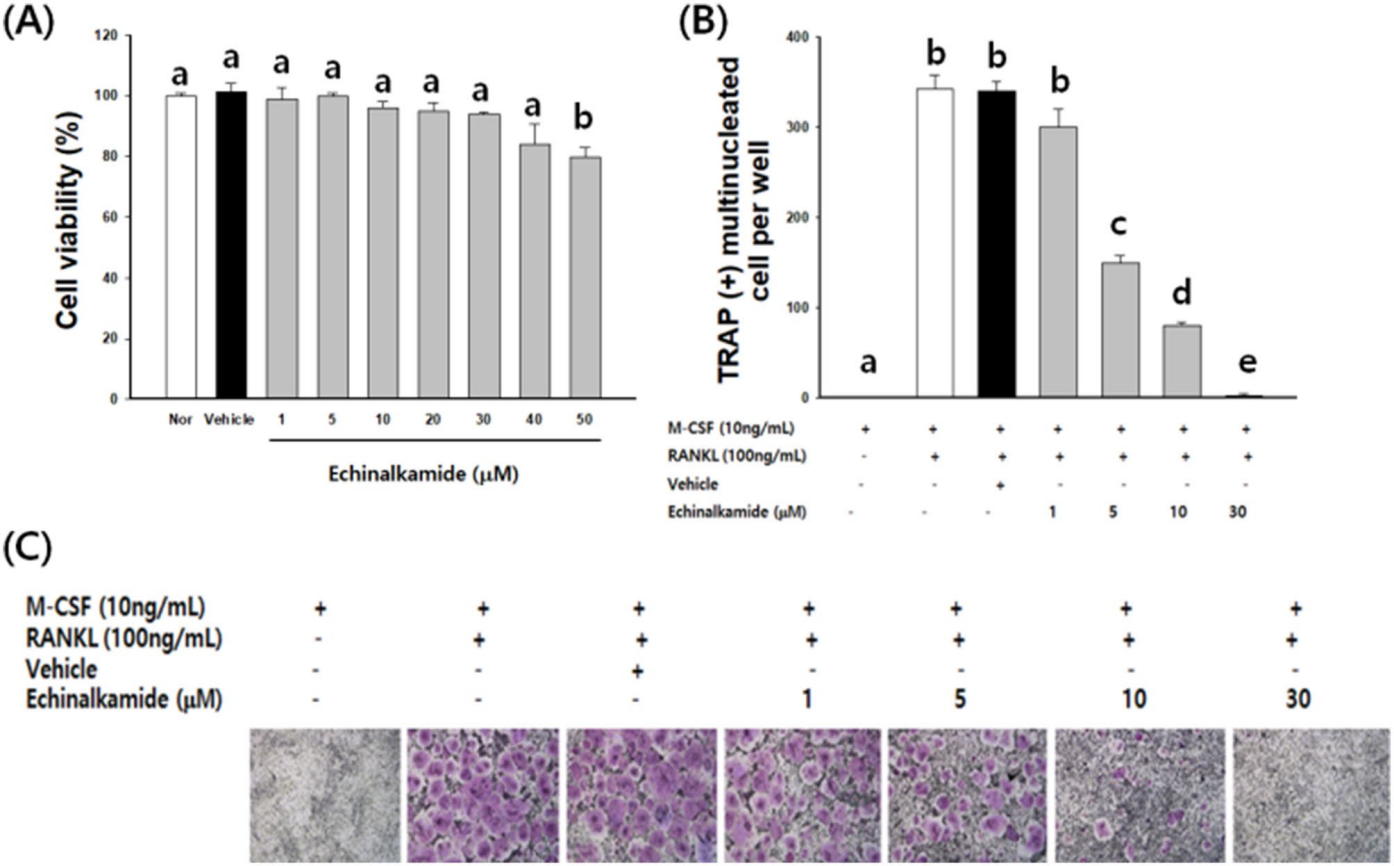
Echinalkamide impairs RANKL-induced osteoclast differentiation. (**A**) The effect of echinalkamide on the viability of BMMs was evaluated using the MTT assay. The BMMs were cultured for 4 days in the presence of RANKL (10 ng/mL) and M-CSF (30 ng/mL) with the indicated concentrations of echinalkamide. (**B**) TRAP-positive MNCs were counted. (**C**) Multinucleated osteoclasts were visualized using TRAP staining. All the data were confirmed by technical replicated (n = 3). The results are presented as the mean ± SD. Values with different letters (a, b, c, d, e) are significantly different one from another (one-way ANOVA followed by Newman–Keuls multiple range test, *p* < 0.05).

BMMs were treated of echinalkamide for 4 days, and viability was evaluated using the MTT assay. Compared to the control group, echinalkamide had any cytotoxic effects on the cells at concentrations less than 30 µM. To exclude the cytotoxic effects, in the following study, echinalkamide concentrations below 30 µM were used for further analysis. (Fig. 5).

### Effect of echinalkamide on osteoclast differentiation in RANKL-stimulated BMMs

**Figure 6:**
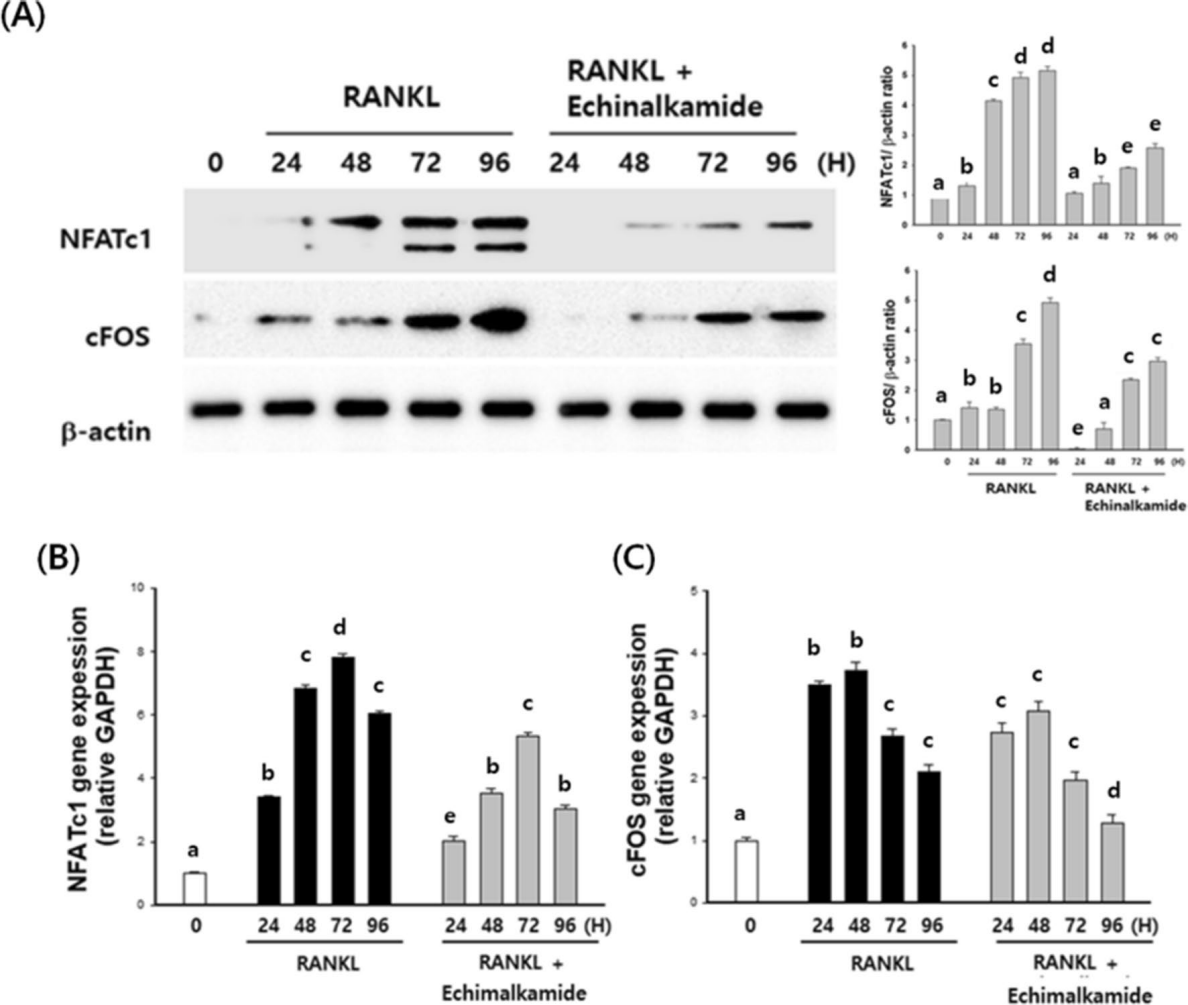
Echinalkamide inhibits the RANKL-mediated expression of c-Fos/NFATc1. BMMs were stimulated with 100 ng/mL RANKL with 30 μM echinalkamide for the indicated periods (0, 24, 48, 72 or 96 h). (**A**) The effect of echinalkamide on the protein expression levels of RANKL-induced transcription factors was evaluated using western blot analysis. Actin was used as the internal control. Full blots are provided in Supplementary Fig. S01. (**B**, **C**) Total RNA was then isolated using easy blue kit, and the mRNA expression levels were evaluated using real-time PCR. GAPDH was used as the internal control. All the data were confirmed by technical replicated (n = 3). The results are presented as the mean ± SD. Values with different letters (a, b, c, d, e) are significantly different one from another (one-way ANOVA followed by Newman-Keuls multiple range test, *p* < 0.05).

To examine the effect of echinalkamide on RANKL-induced osteoclast differentiation, BMMs were treated with various concentrations (1–30 µM) of echinalkamide in the presence of M-CSF or/and RANKL. BMMs without treated to echinalkamide differentiated into TRAP-positive multinucleated cells. Whereas, treated to echinalkamide observed a dose-dependently inhibits in the number of TRAP-positive multinucleated cells (MNCs) (Fig. 6C). The number of TRAP-positive MNCs was significantly decreased when echinalkamide was treated with BMM cells at a concentration of 1–30 μM. The quantification of TRAP-positive MNCs during echinalkamide treatment was from 411 ± 10.1 (0 μM) to 54 ± 9.8 (30 μM) per well. (Fig. 6B).

### Effect of echinalkamide on regulators of osteoclastogenesis

**Figure 7:**
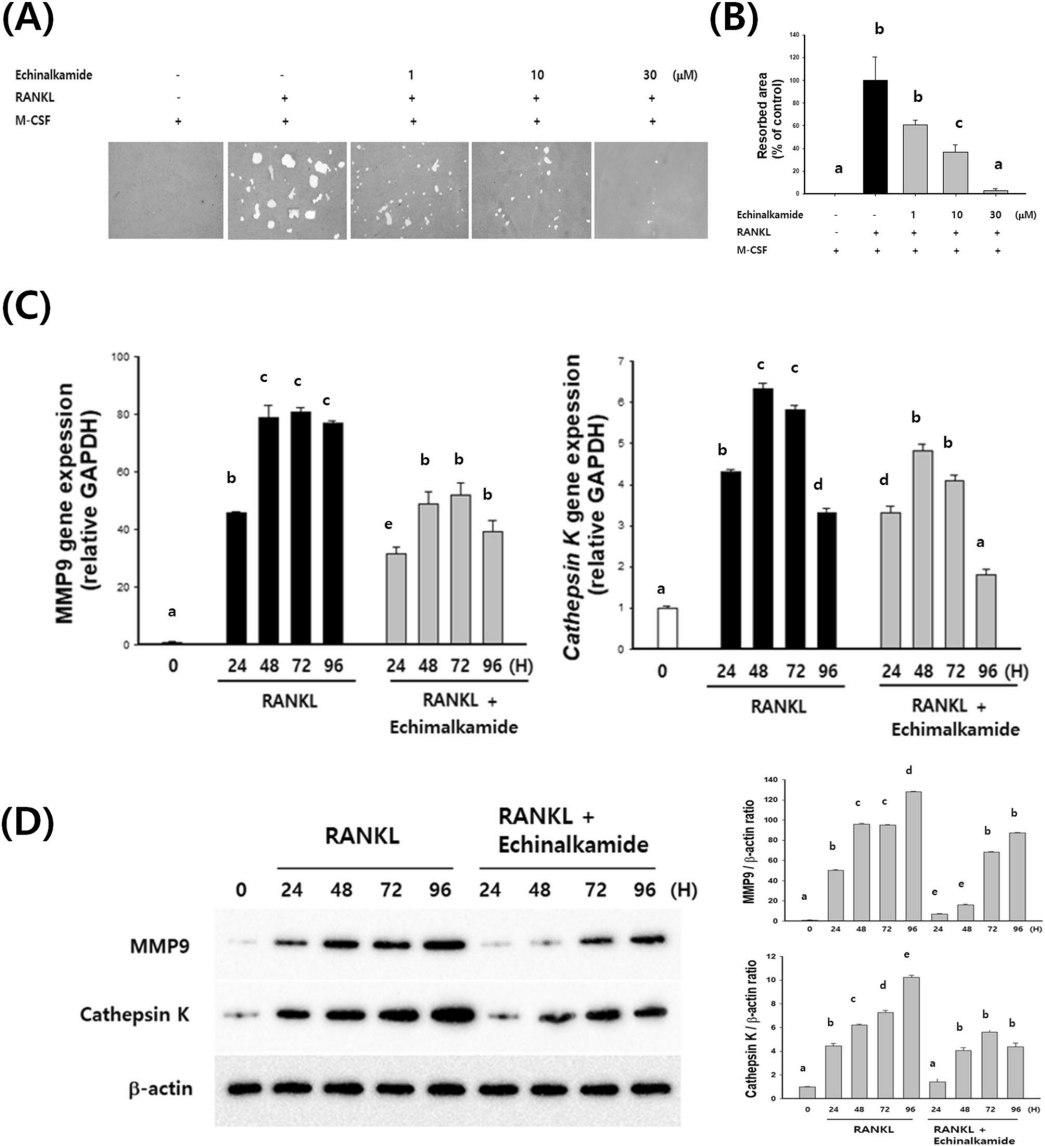
Echinalkamide inhibits the bone resorbing activity of mature osteoclasts. BMMs were stimulated with RANKL (10 ng/mL) and M-CSF (30 ng/mL) in the presence or absence of echinalkamide on mineralized matrix Osteo Assay Surface 96-well plates for 7 days. After that, (**A**) the cells were removed and photographed under a light microscope at the indicated magnification (50×). (**B**) The resorption areas (%) were quantified using the Image J program. (**C**, **D**) The mRNA expression levels of MMP-9 and cathepsin K were analyzed by quantitative real-time PCR. GAPDH was used as the internal control. (**E**) The protein levels of MMP-9 and cathepsin K were analyzed by western blot analysis. Actin was used as the internal control. Full blots are provided in Supplementary Fig. S02. All the data were confirmed by technical replicated (n = 3). The results are presented as the mean ± SD. Values with different letters (a, b, c, d, e) are significantly different one from another (one-way ANOVA followed by Newman–Keuls multiple range test, *p* < 0.05).

We investigated RT-PCR and western blot to measure whether echinalkamide is related to modulator of osteoclastogenesis. Osteoclast precursors were pretreated with echinalkamide and then stimulated with RANKL for different time intervals (6, 12, 24, and 48 h). We found that c-Fos and NFATc1 mRNA and protein levels increased upon treated to RANKL. However, c-Fos and NFATc1 expression was significantly supressed by echinalkamide (Fig. 7A). These results indicated that the inhibitory effects of echinalkamide includes the inhibition of transcription factors such as c-Fos and NFATc1.

### Effect of echinalkamide on bone resorption

BMMs were seeded onto osteoclastic resorption assay plates in induction medium and treated to 1, 10, and 30 μM echinalkamide. As a result, bone resorption was activated by RANKL stimulation. Whereas that the percentage of resorption area decreased after treated to 1 μM echinalkamide, and the resorption was thoroughly suppressed at 30 μM (Fig. 7A, B). Ultimately, the results suggested that treatment with echinalkamide reduced bone resorption in vitro. The RT-PCR and western blot data proved that echinalkamide supressed mRNA and the protein expression of some transcription factors (MMP9 and cathepsin K) associated with cell fusion and osteoclastic bone resorption (Fig. 7C–E).

### Effect of echinalkamide on RANKL-stimulated MAPK and NFkB signaling

**Figure 8:**
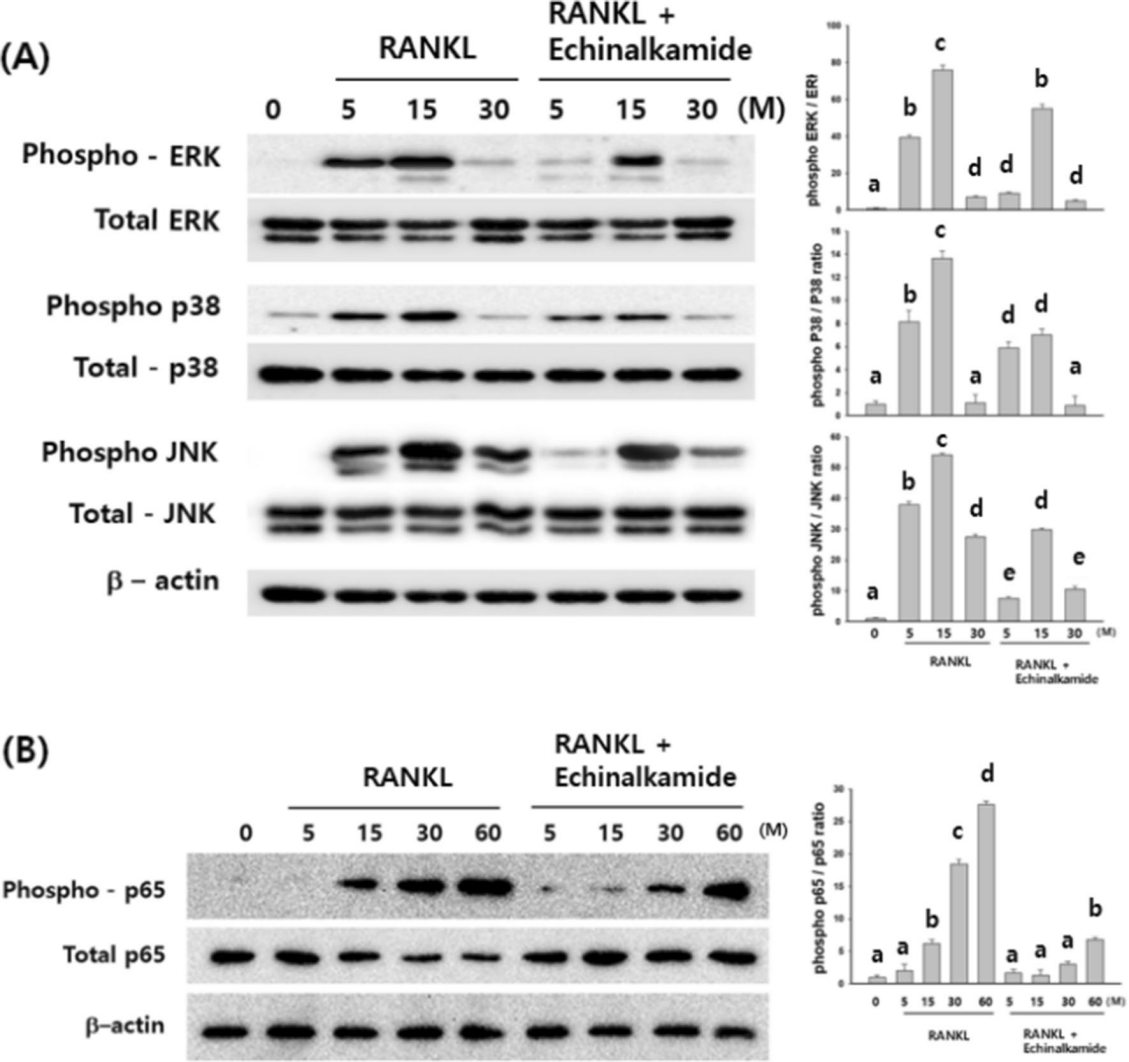
Echinalkamide inhibits the phosphorylation of MAPK. BMMs were stimulated with 100 ng/mL RANKL with 30 μM echinalkamide for the indicated periods (0, 5, 15, 30 or 60 min), (**A**) total and phosphorylation of ERK, p38, JNK and (**B**) NFkB was determined by western blotting and quantified accordingly. Full blots are provided in Supplementary Fig. S03. All the data were confirmed by technical replicated (n = 3). The results are presented as the mean ± SD. Values with different letters (a, b, c, d, e) are significantly different one from another (one-way ANOVA followed by Newman–Keuls multiple range test, *p* < 0.05).

RANKL-stimulated signaling pathways were investigated to demonstrate the underlying molecular mechanisms of echinalkamide inhibitory effects on osteoclast differentiation. JNK, p38, and ERK are members of the MAPKinase and can be activated by RANKL. In the control group, the phosphorylation of ERK, JNK, and p38 peaked within 15 min after RANKL stimulation. However, JNK and ERK phosphorylation was remarkably inhibited after treat with echinalkamide. The quantitative analysis confirmed these observations (Fig. 8).

## Discussion

Bone loss occurs when cycle of removal of old bone faster than the deposition of new bone^27,28^. The loss of bone occurs with age, poor diet, excessive vitamin A levels, low levels of sex hormones, bed rest or inactivity, smoking, and excessive consumption of alcohol and caffeine^29–31^. Bone loss can contribute to decrease of bone density, bone weakness, and ultimately osteoporosis. Osteoporosis is the result of bone loss and accumulative bone structure damage that can break the bone with minimal trauma. These bone loss or osteoporosis can be prevented through appropriate nutrition, physical activity and, if necessary, appropriate treatment^32,33^.

The existing approved drugs for osteoporosis include bisphosphonates, ecombinant human parathyroid hormone (PTH), hormone replacement therapy (HRT), selective estrogen receptor modulators (SERMS), and denosumab^29,31^. However, these osteoporosis treatments can cause serious side effects such as hypocalcemia, stroke, heart attack, thrombosis and osteonecrosis, and alternative therapies are needed to prevent this. Natural compounds derived from medicinal plants and foods are interested in developing effective and safe treatments for bone diseases^34–36^. According to Che et al., there have been many in vitro and in vivo biological and pharmacological studies demonstrating a wide variety of natural products such as *Carthami Flos*, *Cistanches Herba*, *Echinacea* spp*.*, *Cordyceps sinensis*, *Dipsaci Radix*, *Drynaria Rhizoma*, *Ecliptae Herba*, *Epimedii Folium*, *Morindae Officinalis Radix*, *Puerariae lobatae Radix*, and *Sophorae Fructus* that have potential beneficial effects for the maintenance or promotion of bone health^37^. Many studies have found natural compounds derived from plants that are effective in inhibiting osteoporosis, including terpenoids^38,39^, flavonoids^40,41^, glucosides^42^, polyphenols^43,44^, limonoids^34,45^, lignans^46,47^, alkaloids^48,49^, anthraquinones^50^, and coumarin^51^.

Many osteoclast suppressors are associated with anti-inflammatory and antioxidant^52,53^. According to Aarland et al., *E. purpurea* has already been studied for its effect on anti-inflammatory and anti-oxidant activity^54^. Therefore it was hypothesized that it may also be effective on inflammation-related osteoclastogenesis. Fractionation of adventitious roots of *E. purpurea* using various chromatographic techniques yielded three new compounds together with eight known compounds. We first performed an antioxidant and anti-inflammatory activity, in order to select a new compound effective in inhibiting osteoclasts. Echinalkamide (compound **1**, undeca-2Z-4E-diene-8,10-diynoic acid isobutylamide), which has the highest antioxidant and anti-inflammatory effects among the new compounds was selected. In the present study, we investigated the potential of echinalkamide as an osteoporosis treatment and its underlying mechanism.

Most disorders of bone metabolism induce activation of osteoclasts^28,55^. As a result, bone resorption goes beyond bone formation, leading to pathological bone resorption activity and causing osteopenia, which increases the risk of fracture. Osteoclasts are important target cells for osteoporosis treatment^29,56^.

Osteoclasts play an important role in the pathological destruction of bone. The differentiation of osteoclasts differentiates into osteoclasts that resorpsion bone through several stages. When RANKL is bound to RANK expressed in monocytes or macrophages, it differentiates into tartrate-resistant acid phosphatase (TRAP) positive cells. The binding of RANKL to RANK on the surface of osteoclasts induces intracellular signal transduction pathways such as NF-κB, MAPK and calcium rash to increase expression of NFATc1, an essential transcription factor involved in the formation of osteoclasts.^55,57,58^.

In the present study, echinalkamide has been demonstrated to be an effective inhibitor of osteoclastogenesis in vitro in terms of number and area reduction of TRAP-positive multinucleated cells. It has been reported that embryonic stem cells deficient in NFATc1 do not differentiate into osteoclasts in response to RANKL stimulation^55,58,59^. Our study has demonstrated that echinalkamide supresses RANKL-induced NFATc1 activation. We further investigated its effect on RANKL activation of MAPKs.

The binding of RANKL and RANK results in the congestion of the cell RANK domain with TNT receptor-associated factor 6 (TRAF6). This activates transcription factors such as nuclear factor kappa B (NF-κB), activator protein-1 (AP1) and NFATc1, which are the subsequent genes of TRAF6, resulting in activation of p38, JNK and extracellular-signal regulated kinases (ERK). Activation is carried out at the protein level through phosphorylation processes including mitogen-activated protein kinases (MAPKs) and Phosphatidyl-inositol-3-kinase (PI3K)/Akt pathway^60,61^. Many studies have revealed that MAPKs can be stimulated by RANKL stimulation and are associated with osteoclastogenesis^62–65^. RANKL stimulated ERK, JNK, and p38 through activation of MEK1/2, MKK7, and MKK6 to induce activation of their downsignal targets such as c-Fos, AP-1 transcription factors, and microphthalmia-associated transcription factor (MITF) in osteoclast precursors, respectively^62,66^. p38 is important in the early stages of osteoclast generation because it regulates MITF. ERK was found to be able to induce the expression of downstream molecules that induce osteoclast generation, and blockade of the ERK pathway was shown to inhibit osteoclast formation^67,68^. Dominant-negative JNK prevents RANKL-induced osteoclastogenesis. JNK plays an important role in osteoclast generation, according to studies using knockout models^69^. We observed that echinalkamide inhibits the MAPK signaling pathway by suppressing phosphorylation of p-38, JNK and ERK. Several of evidence surggest that both NF-κB and c-Fos serve as downstream targets of TRAF6 and play important roles in NFATc1 activation. NF-κB is important for RANKL-mediated induction of NFATc1 in the early stages of osteoclastogenesis. After RANKL stimulation for 24 h, AP-1 containing c-FOS is recruited to the NFATc1 promoter and contributes to the automatic amplification of NFATc1. NFATc1 increases expression of osteoclast-specific genes TRAP, DC-STAMP, and cathepsin K^55,70^. Our results showed that echinalkamide inhibited the RANKL-induced expression of NFATc1 and c-FOS.

NFATc1 is a master transcription factor be demanded for osteoclastogenesis and controlled of marker genes, including TRAP, cathepsin K, DC-STAMP, and MMP-9, which are indispensable for osteoclastic fusion and resorption^55,71^. The presence of echinalkamide attenuated the MAPKinase pathway, protein induction and transcriptional activity of NFATc1. The marker gene, cathepsin K and MMP9, was also significantly reduced. These results suggest that the effect of echinalkamide on osteoclast formation may be partly due to mitigation of the MAPK signaling pathway and subsequent mitigation of NFATc1 induction.

Studies have shown that echinalkamide inhibits osteoclast formation and osteoclast function in vitro through inhibition of MAPK signaling pathway and NFATc1. Therefore, echinalkamide can be used as a potential therapeutic agent for osteoclast-related diseases.

**Figure 9:**
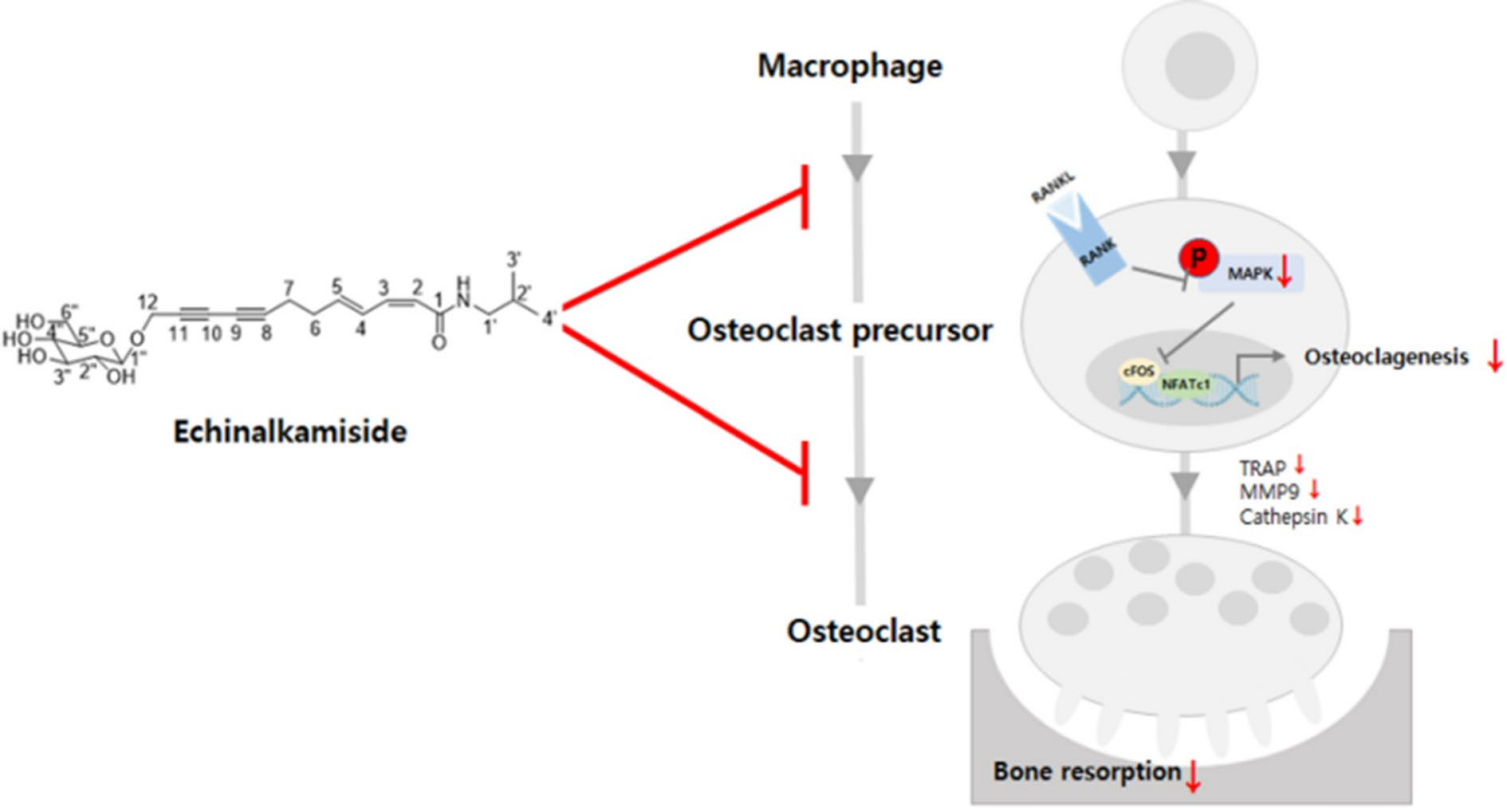
Summary of the effects of *Echinalkamide* against osteoclastogenesis and bone.

Taken together, our present study demonstrated that adventitious root cultures of *E. purpurea* can be used not only for securing materials but also for the discovery of new compounds. In addition, the newly isolated compounds might contribute to the anti-inflammatory effect of E. purpurea. Among them, studies have shown that echinalkamide inhibits osteoclast formation and osteoclast function in vitro through inhibition of MAPK signaling pathway and NFATc1. Echinalkamide can be used as a treatment for osteoclast-related diseases as well as anti-inflammatory agents (Fig. 9).

## Supplementary information

Supplementary information
