# Supplementary material for: Effect of echinalkamide identified from Echinacea purpurea (L.) Moench on the inhibition of osteoclastogenesis and bone resorption
Source: Sci Rep. 2020 Jul 2;10:10914. doi: 10.1038/s41598-020-67890-x (PMC7331694; doi:10.1038/s41598-020-67890-x)
Supplement: Supplementary file 1 — Supplementary information [file 41598_2020_67890_MOESM1_ESM.docx]

**Effect of echinalkamide identified from Echinacea purpurea (L.) Moench on the inhibition of osteoclastogenesis and bone resorption**

Bo Yoon Chang^1+^, Seul Ki Lee ^2+^, Da Eun Kim^1^, Jin Hye Bae^1^, Thanh Tam Ho^2^, So-Young Park^3^, Mi Kyeong Lee ^2*^, Sung Yeon Kim ^1*^

*^1^ Institute of Pharmaceutical Research and Development, College of Pharmacy, Wonkwang University, Iksan, Jeonbuk 54538, South Korea.*

*^2^ College of Pharmacy, Chungbuk National University, Cheongju 28160, Republic of Korea*

*^3^ Department of Horticultural Science, Chungbuk National University, Cheongju 28644, Republic of Korea*

**List of Supporting Information**

**Figure S1.** ^1^H-NMR spectrum of compound **1** (500 MHz, methanol-*d_4_*) ···································2

**Figure S2.** ^13^C-NMR spectrum of compound **1** (125 MHz, methanol-*d_4_*) ··································2

**Figure S3.** HSQC spectrum of compound **1** (125 MHz, methanol-*d_4_*) ······································3

**Figure S4.** HMBC spectrum of compound **1** (125 MHz, methanol-*d_4_*) ······································3

**Figure S5.** HR-ESI-MS data of compound **1**····································································4

**Figure S6.** ^1^H-NMR spectrum of compound **2** (400 MHz, methanol-*d_4_*) ···································4

**Figure S7.** ^13^C-NMR spectrum of compound **2** (100 MHz, methanol-*d_4_*) ··································5

**Figure S8.** HSQC spectrum of compound **2** (100 MHz, methanol-*d_4_*) ······································5

**Figure S9.** HMBC spectrum of compound **2** (100 MHz, methanol-*d_4_*) ······································6

**Figure S10.** HR-ESI-MS data of compound **2**···································································6

**Figure S11.** ^1^H-NMR spectrum of compound **3** (500 MHz, CDCl_3_) ········································7

**Figure S12.** ^13^C-NMR spectrum of compound **3** (125 MHz, CDCl_3_) ·······································7

**Figure S13.** HSQC spectrum of compound **3** (125 MHz, CDCl_3_) ···········································8

**Figure S14.** HMBC spectrum of compound **3** (125 MHz, CDCl_3_) ··········································8

**Figure S15.** HR-ESI-MS data of compound **3**···································································9

**
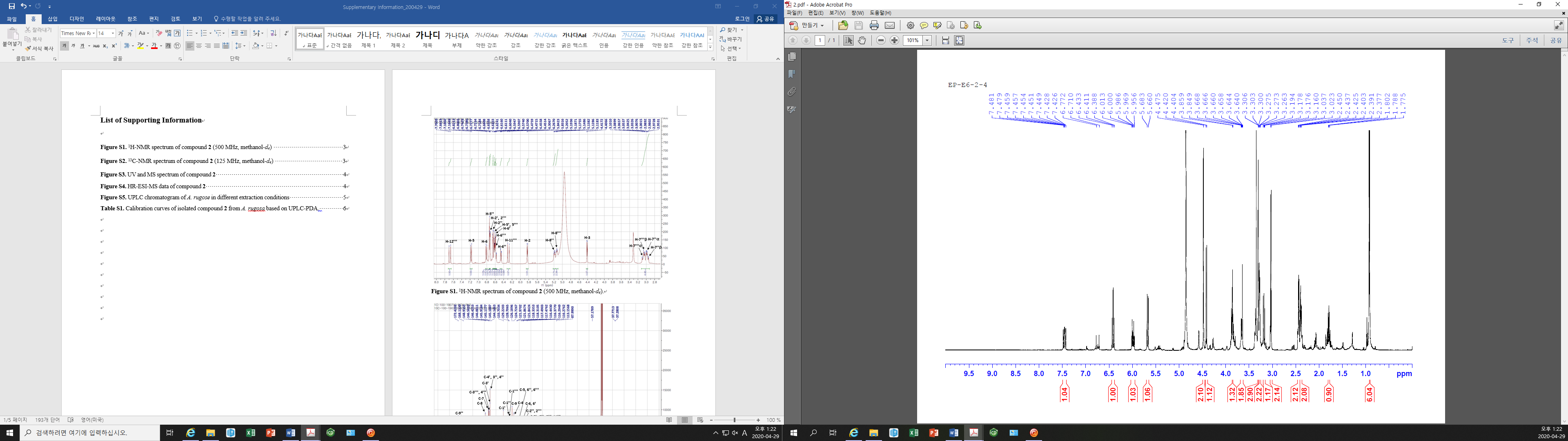
 Figure S1.** ^1^H-NMR spectrum of compound **1** (500 MHz, methanol-*d_4_*) **
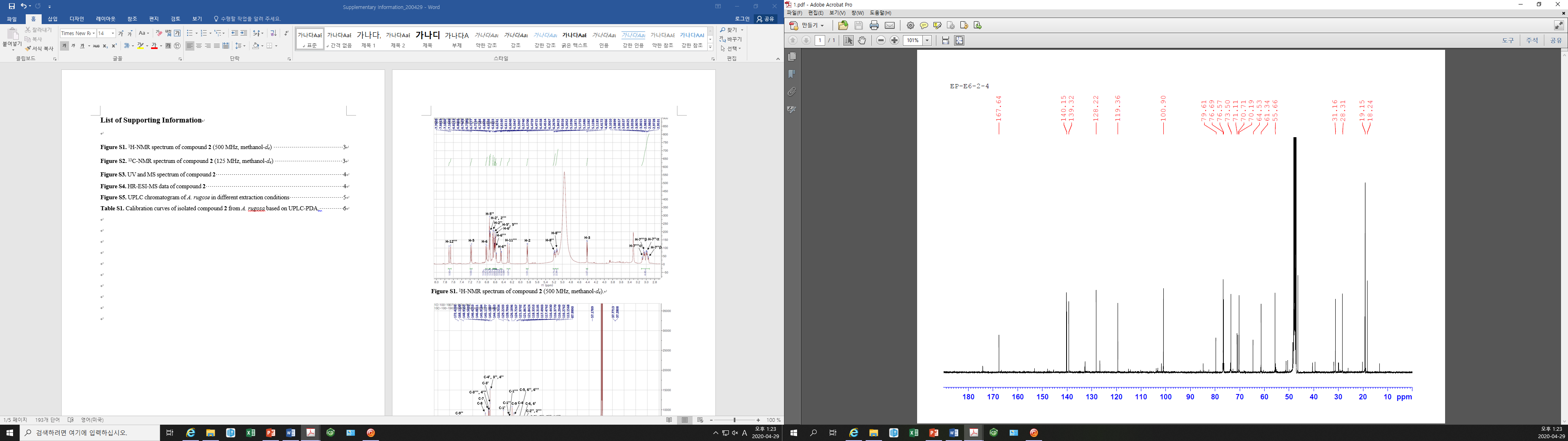
 Figure S2.** ^13^C-NMR spectrum of compound **1** (125 MHz, methanol-*d_4_*) **
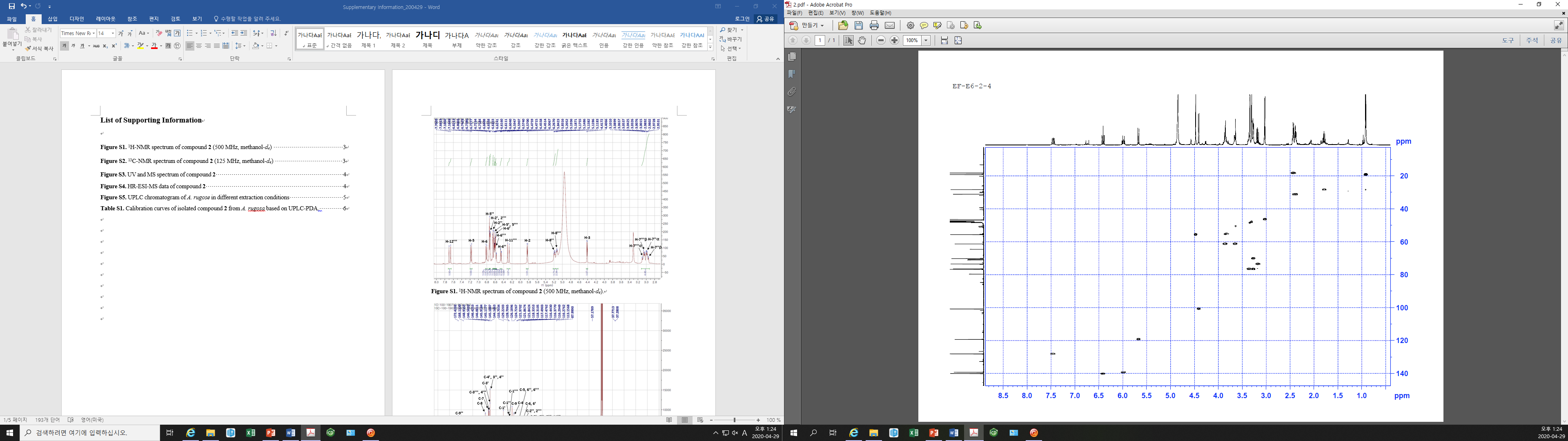
 Figure S3.** HSQC spectrum of compound **1** (125 MHz, methanol-*d_4_*)
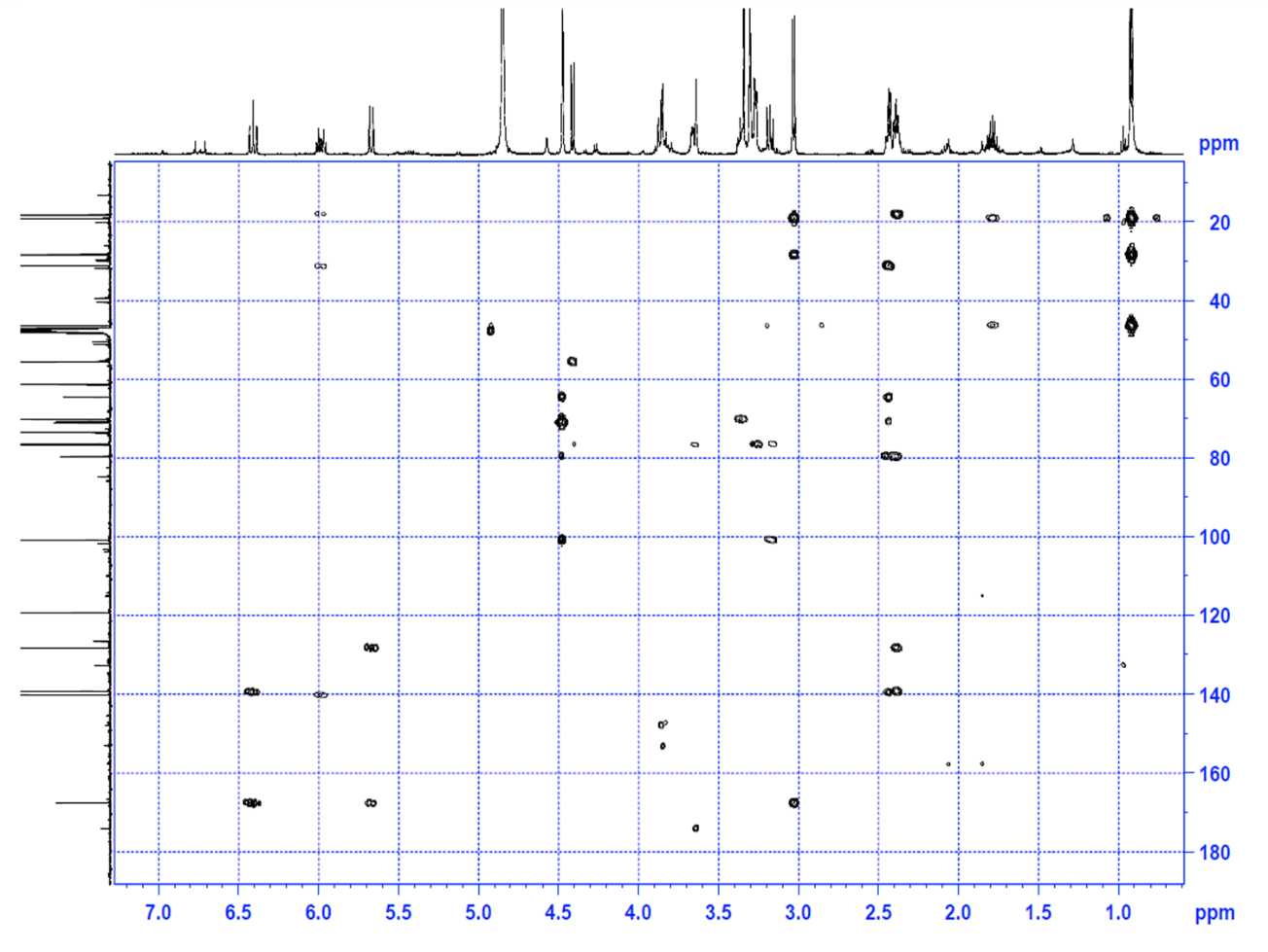
 **Figure S4.** HMBC spectrum of compound **1** (125 MHz, methanol-*d_4_*) **
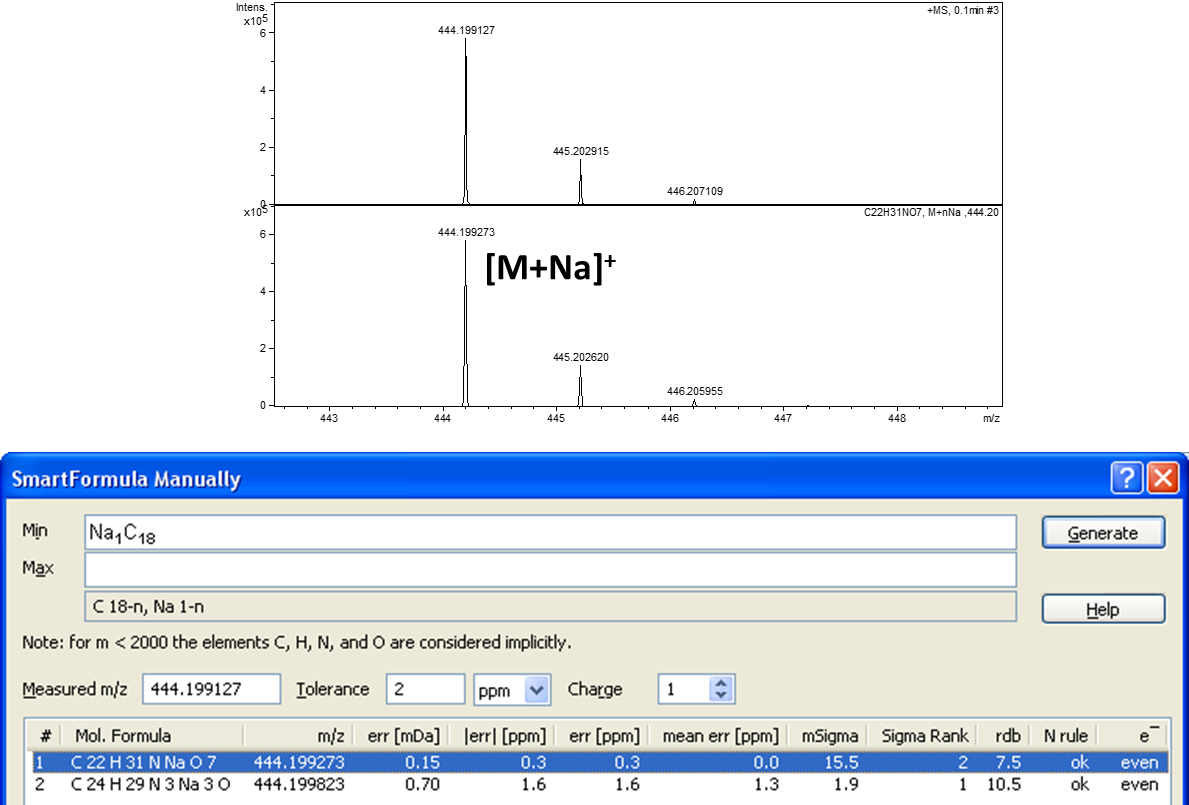
 Figure S5.** HR-ESI-MS data of compound **1
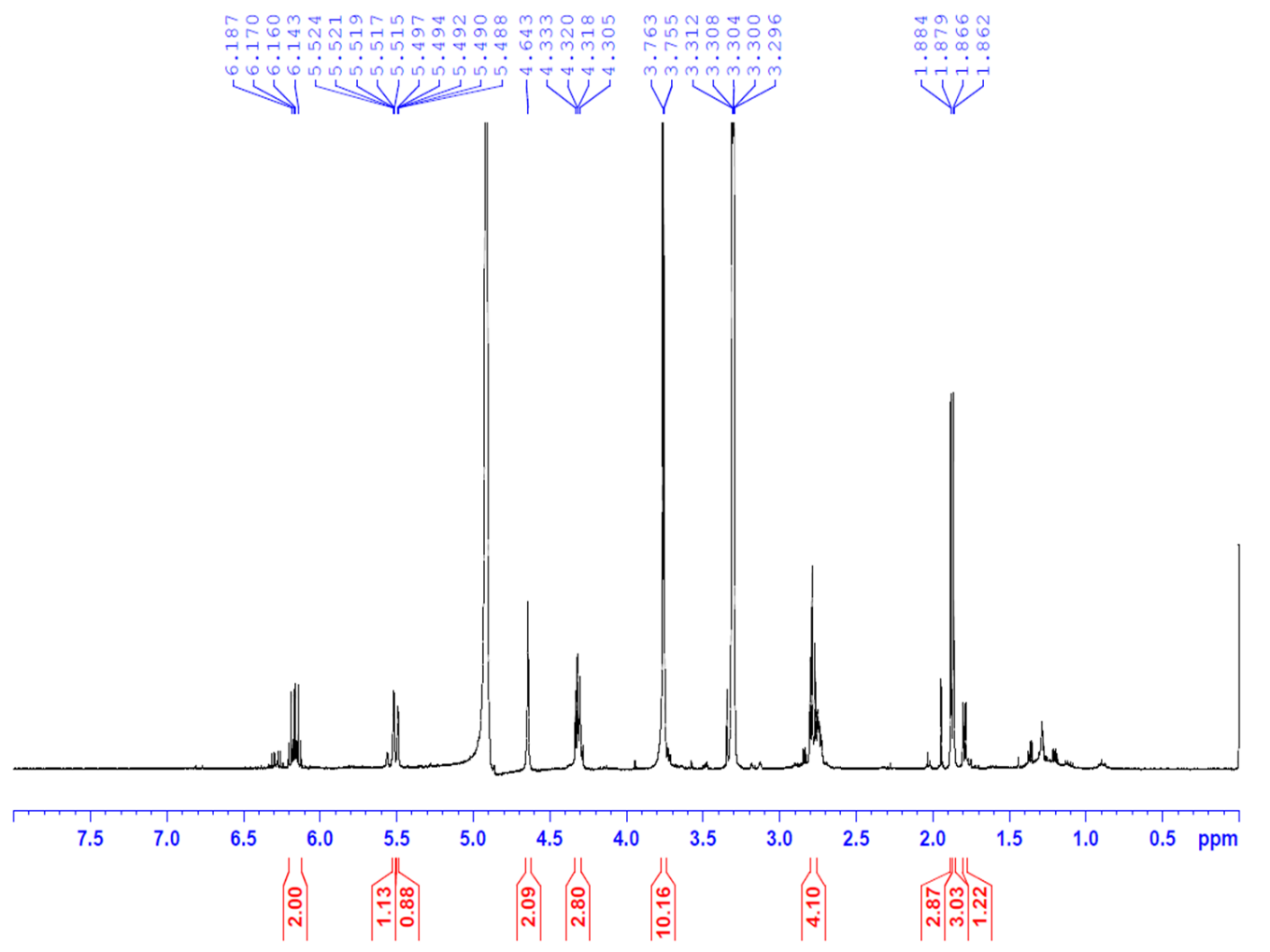
 Figure S6.** ^1^H-NMR spectrum of compound **2** (400 MHz, methanol-*d_4_*) **
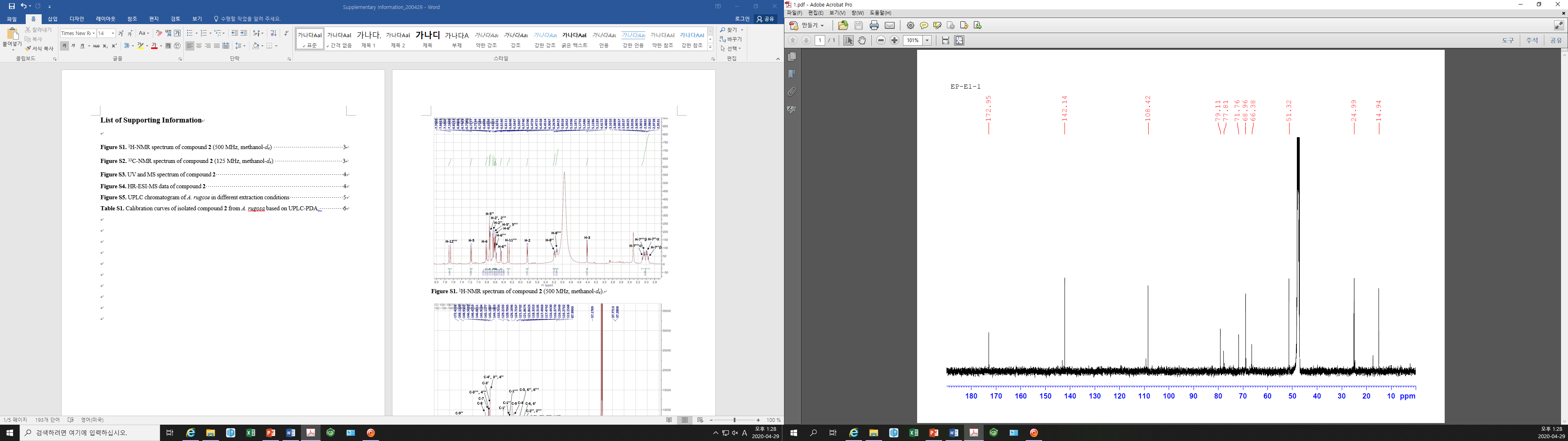
 Figure S7.** ^13^C-NMR spectrum of compound **2** (100 MHz, methanol-*d_4_*)
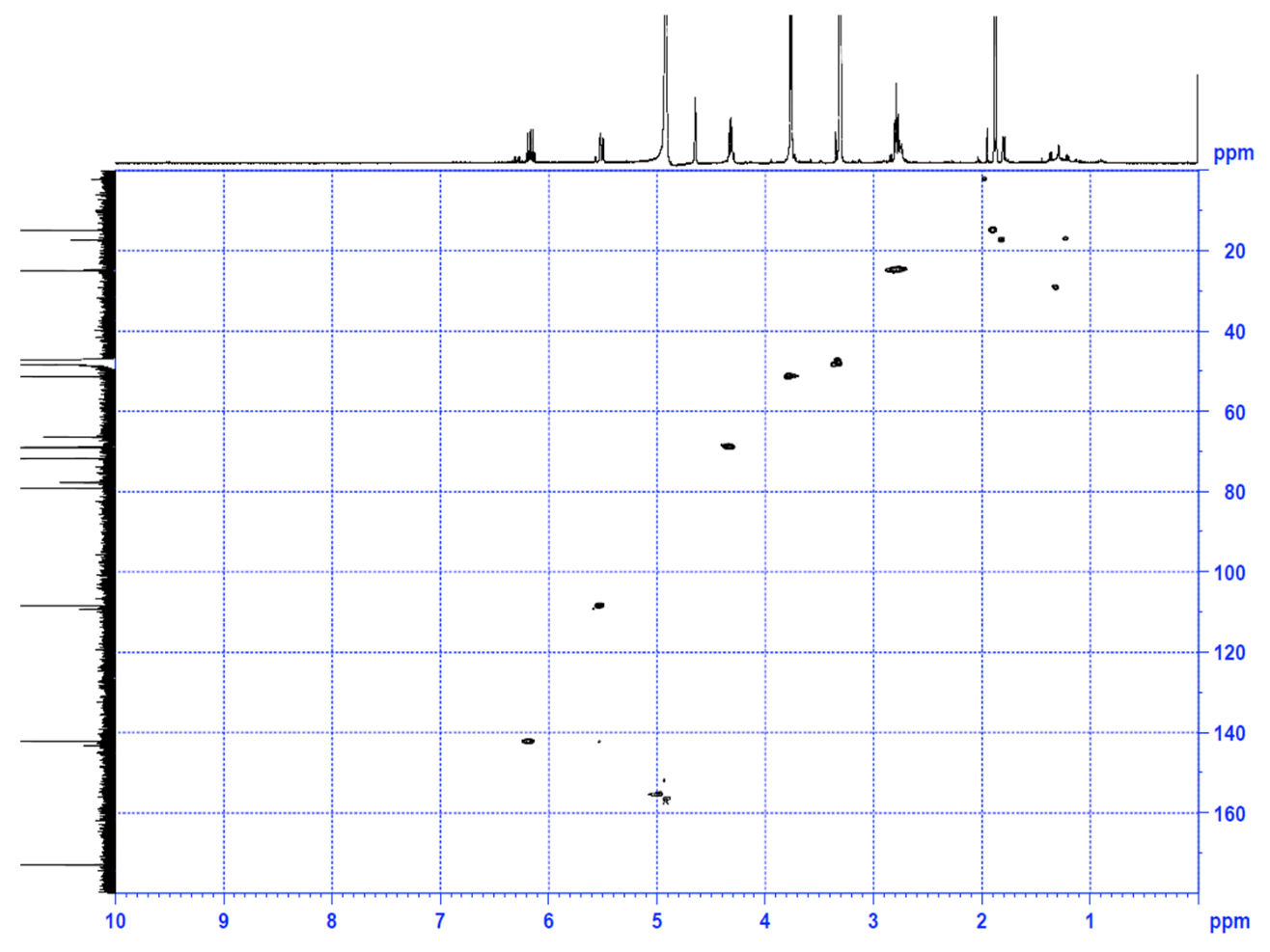
 **Figure S8.** HSQC spectrum of compound **2** (100 MHz, methanol-*d_4_*)
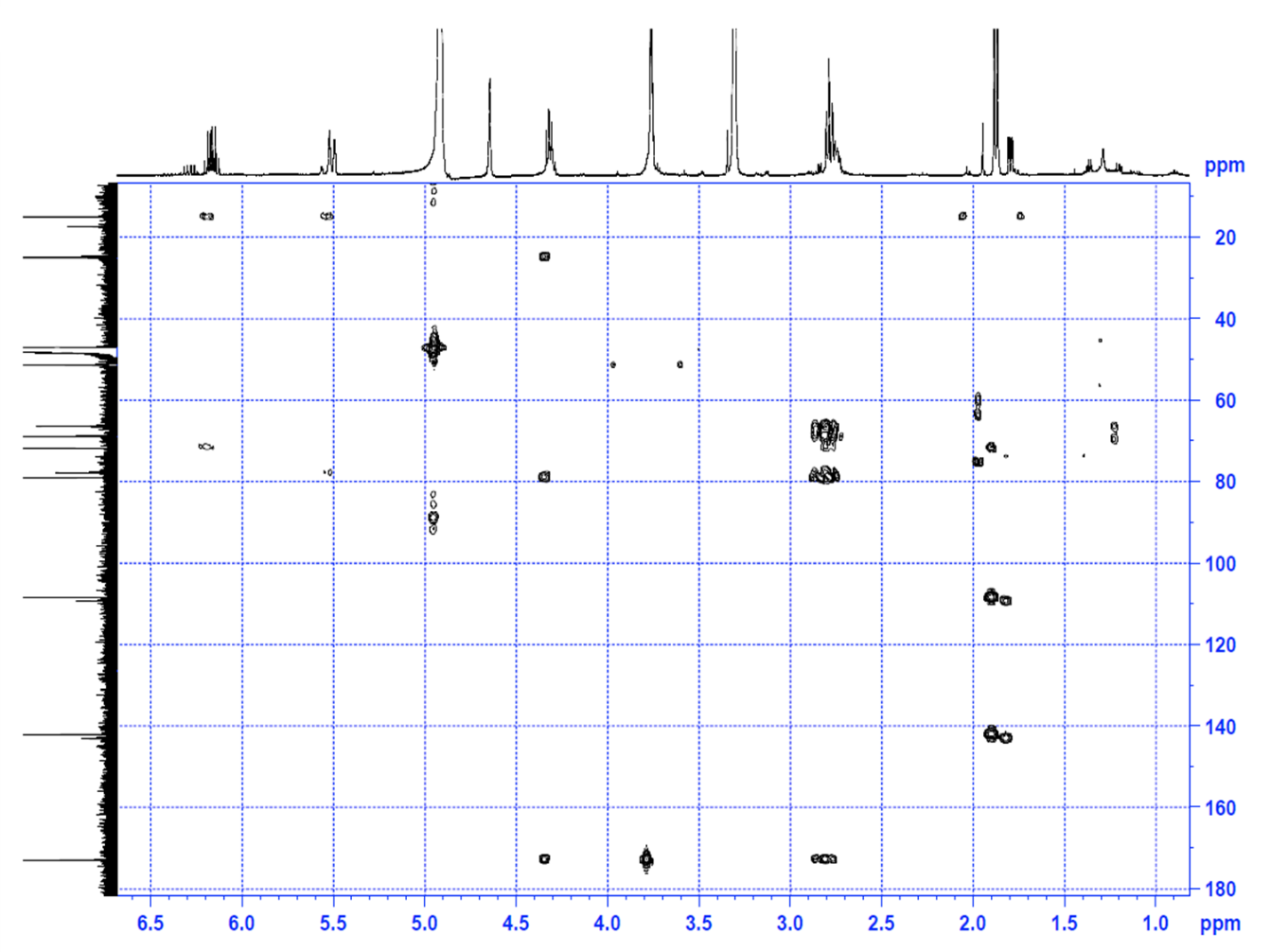
 **Figure S9.** HMBC spectrum of compound **2** (100 MHz, methanol-*d_4_*) **
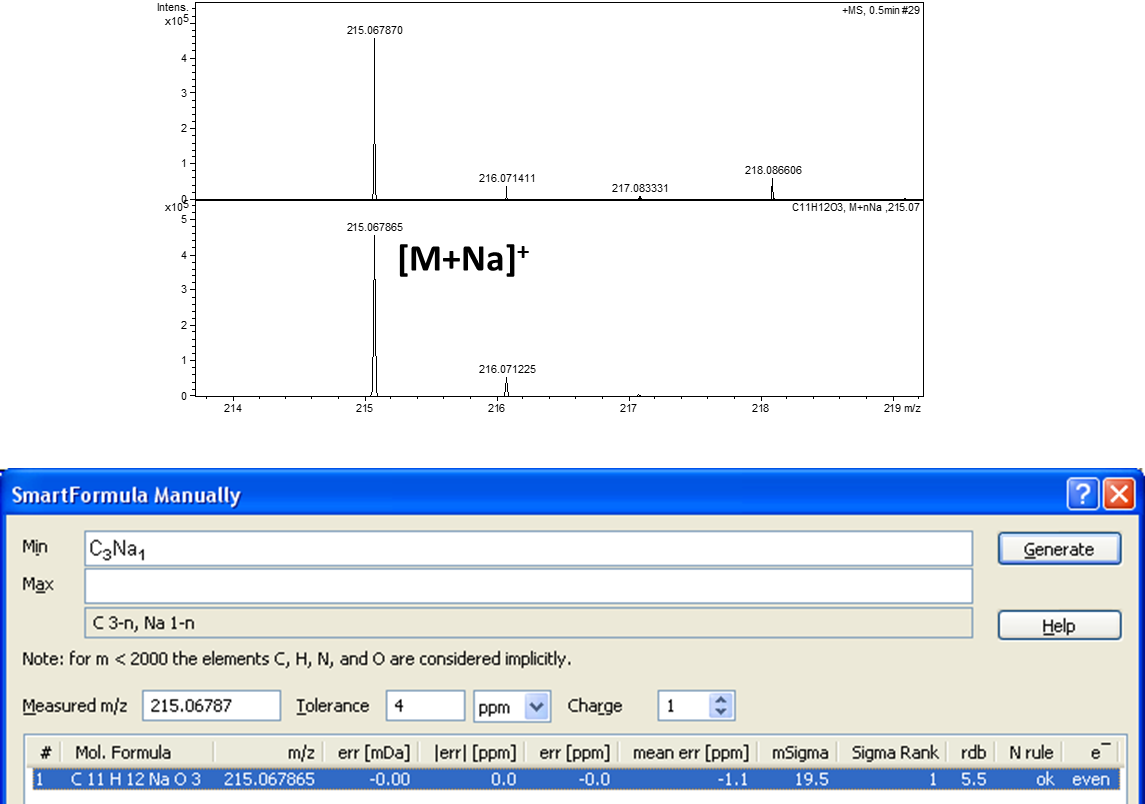
 Figure S10.** HR-ESI-MS data of compound **2
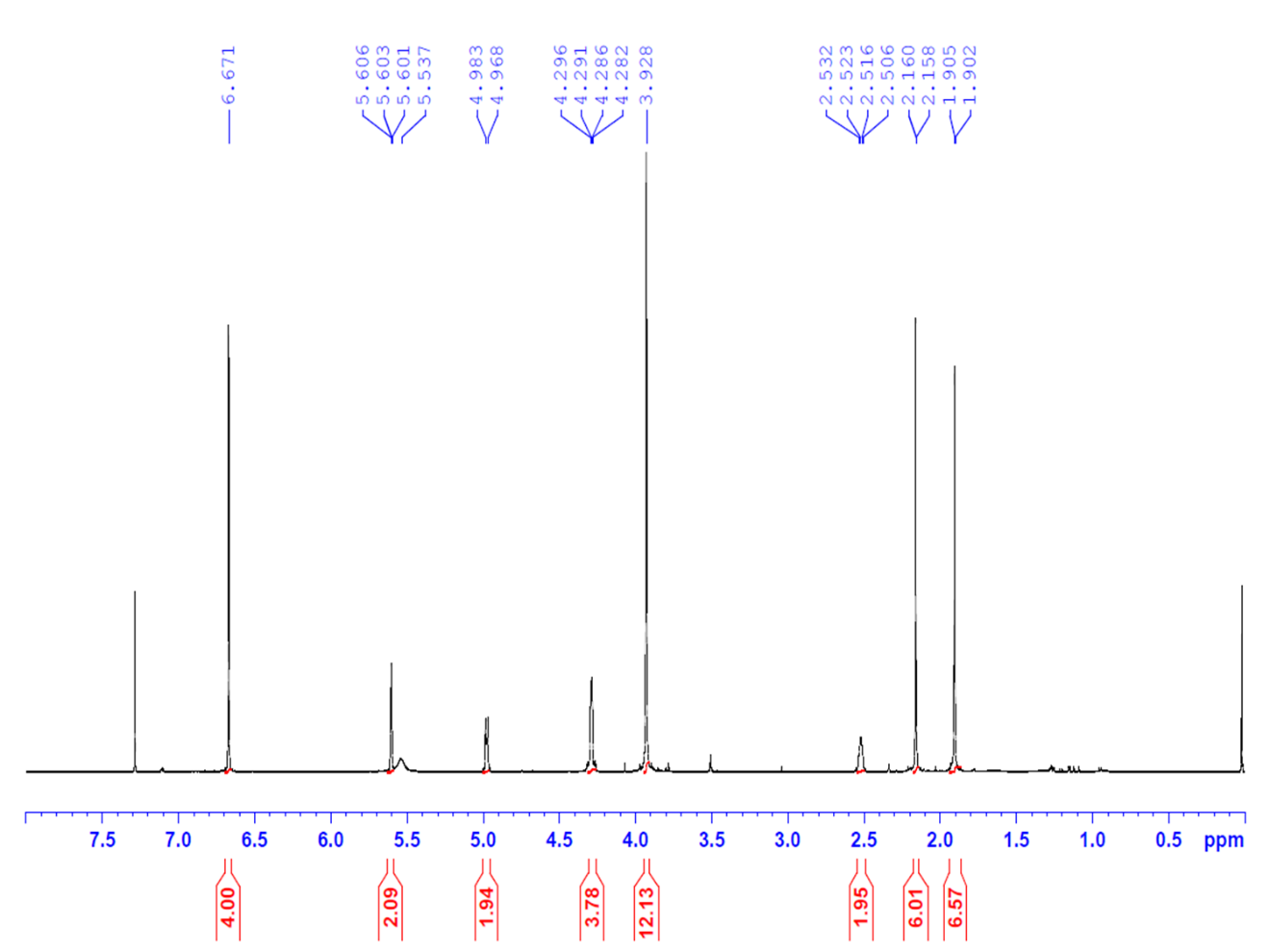
Figure S11.** ^1^H-NMR spectrum of compound **3** (500 MHz, CDCl_3_)
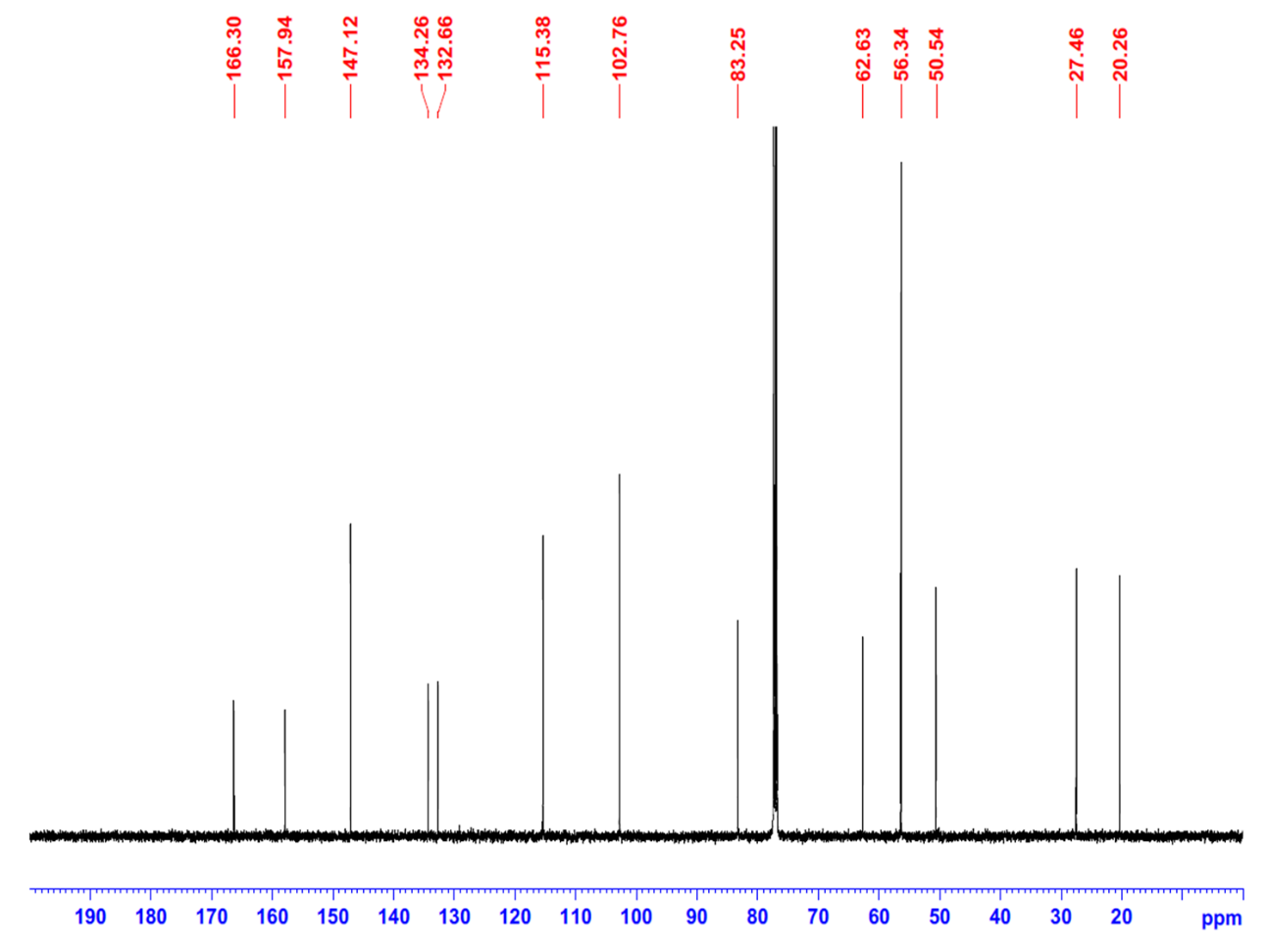
 **Figure S12.** ^13^C-NMR spectrum of compound **3** (125 MHz, CDCl_3_) **
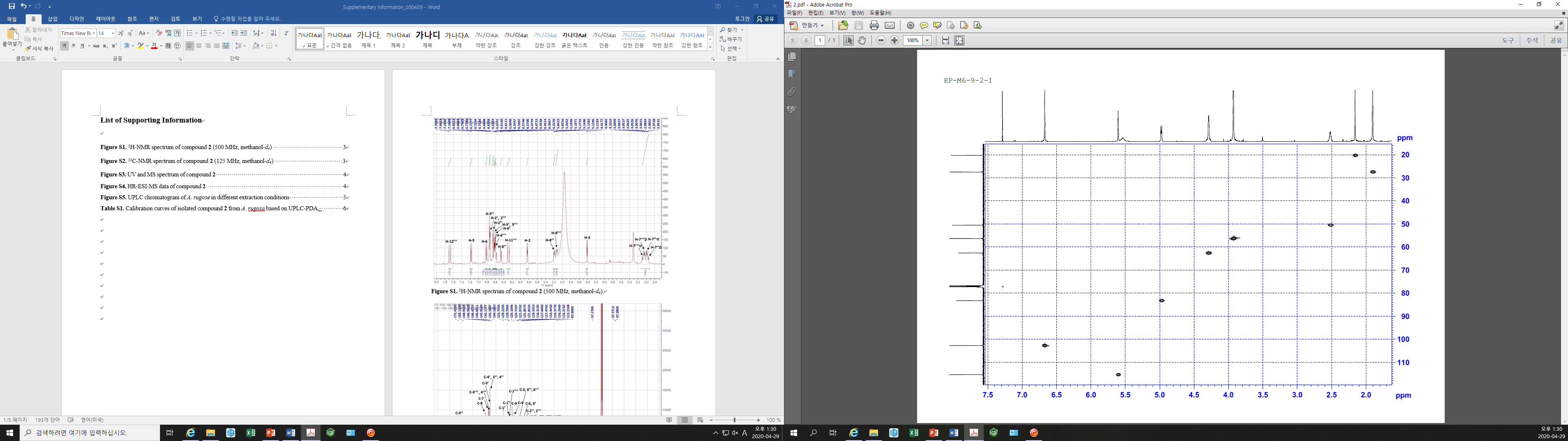
 Figure S13.** HSQC spectrum of compound **3** (125 MHz, CDCl_3_)
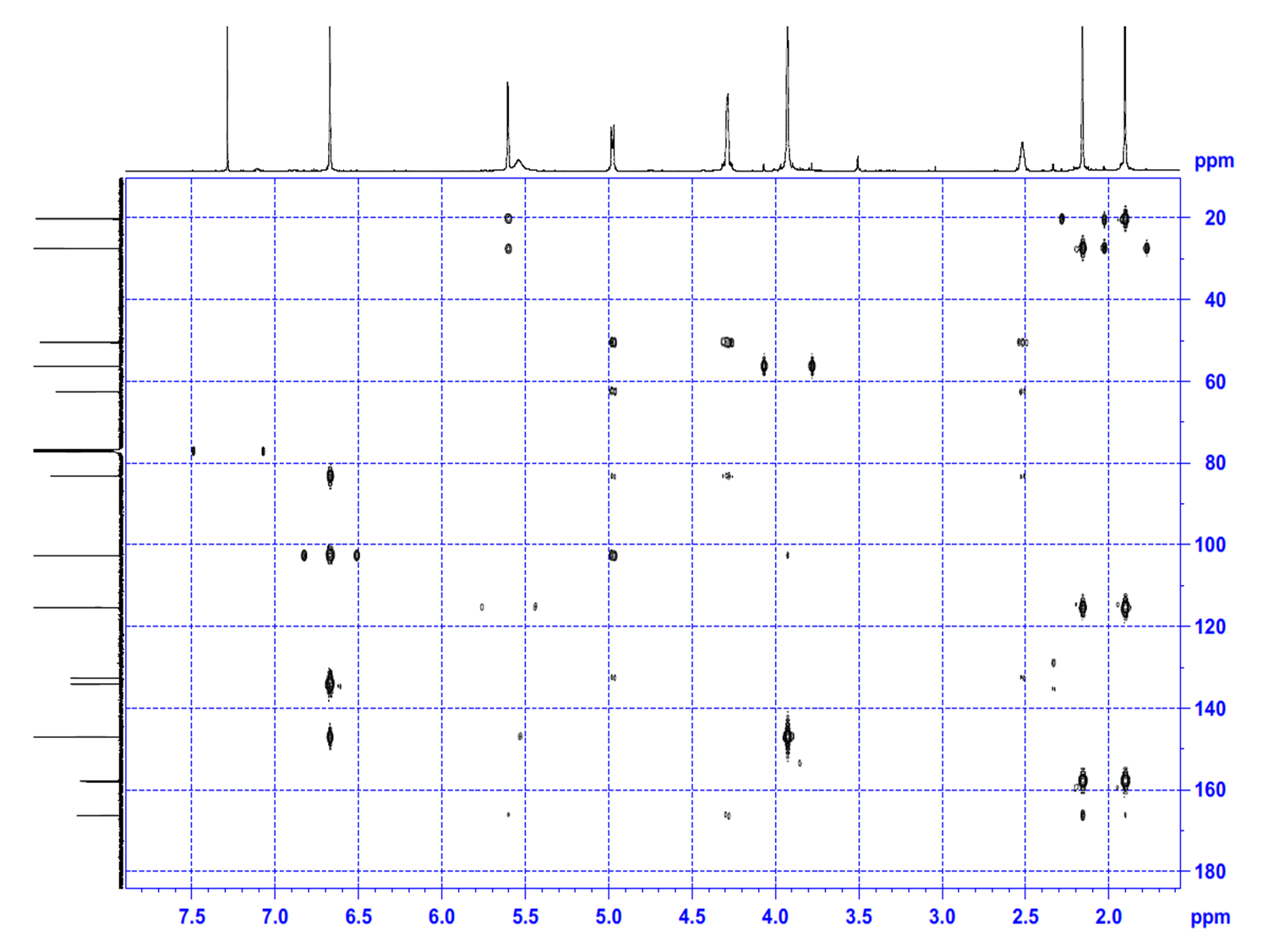
 **Figure S14.** HMBC spectrum of compound **3** (125 MHz, CDCl_3_)

**
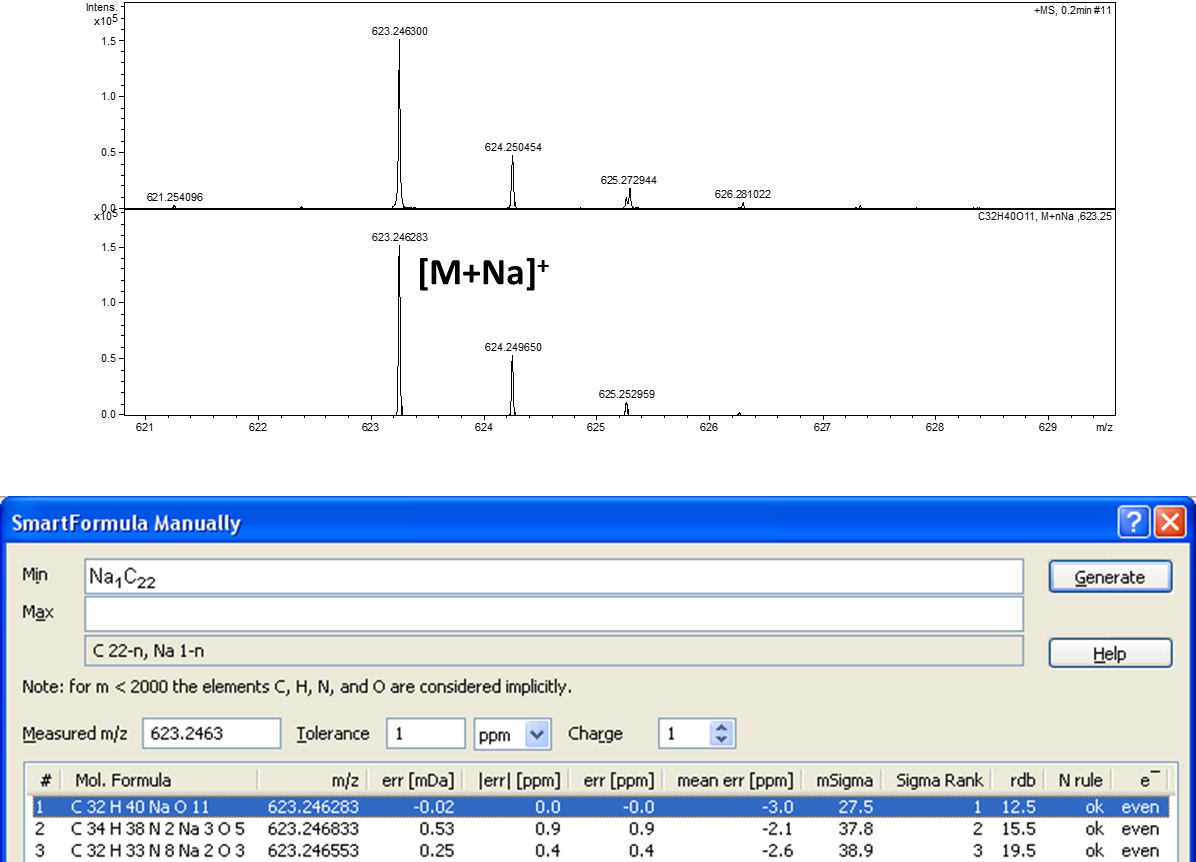
Figure S15.** HR-ESI-MS data of compound **3**
